# Scale-free dynamics in the core-periphery topography and task alignment decline from conscious to unconscious states

**DOI:** 10.1038/s42003-023-04879-y

**Published:** 2023-05-09

**Authors:** Philipp Klar, Yasir Çatal, Robert Langner, Zirui Huang, Georg Northoff

**Affiliations:** 1 Medical Faculty, C. & O. Vogt-Institute for Brain Research, Heinrich Heine University of Düsseldorf, Düsseldorf, Germany; 2 The Royal’s Institute of Mental Health Research & University of Ottawa. Brain and Mind Research Institute, Centre for Neural Dynamics, Faculty of Medicine, University of Ottawa, Ottawa, 145 Carling Avenue, Rm. 6435, Ottawa, ON K1Z 7K4 Canada; 3 Institute of Systems Neuroscience, Heinrich Heine University Düsseldorf, Düsseldorf, Germany; 4 Institute of Neuroscience and Medicine, Brain & Behaviour (INM-7), Research Centre Jülich, Jülich, Germany; 5 Department of Anesthesiology, University of Michigan Medical School, Ann Arbor, MI 48109 USA; 6 Center for Consciousness Science, University of Michigan Medical School, Ann Arbor, MI 48109 USA; 7 Centre for Cognition and Brain Disorders, Hangzhou Normal University, Tianmu Road 305, Hangzhou, Zhejiang Province 310013 China

**Keywords:** Consciousness, Perception

## Abstract

Scale-free physiological processes are ubiquitous in the human organism. Resting-state functional MRI studies observed the loss of scale-free dynamics under anesthesia. In contrast, the modulation of scale-free dynamics during task-related activity remains an open question. We investigate scale-free dynamics in the cerebral cortex’s unimodal periphery and transmodal core topography in rest and task states during three conscious levels (awake, sedation, and anesthesia) complemented by computational modelling (Stuart-Landau model). The empirical findings demonstrate that the loss of the brain’s intrinsic scale-free dynamics in the core-periphery topography during anesthesia, where pink noise transforms into white noise, disrupts the brain’s neuronal alignment with the task’s temporal structure. The computational model shows that the stimuli’s scale-free dynamics, namely pink noise distinguishes from brown and white noise, also modulate task-related activity. Together, we provide evidence for two mechanisms of consciousness, temporo-spatial nestedness and alignment, suggested by the Temporo-Spatial Theory of Consciousness (TTC).

## Abstract (web summary)

Investigation of scale-free brain dynamics via rest-task modulation across awake, sedative, and anesthetic states of consciousness shows that brain dynamics lose alignment to the task’s temporal structure in anesthesia as in wakefulness.

## Introduction

Scale-free, fractal, or self-similar dynamics are ubiquitous in nature^1,2^ and in the human organism, including its nervous system^3–5^. Statistical self-similarity describes objects in space or processes in time where smaller pieces resemble the statistics of the whole instead of being an exact geometrical copy as found in mathematically created fractals^6^. Paradigmatic examples of scale-free physiological processes measured in time-series are the voltage across the cell membrane of T-lymphocytes^7^, currents through ion channels^8^, heart rate variability^9–11^, blood flow^12^, volumes of breaths^13^, and functional magnetic resonance (fMRI) recordings of human brain activity^14–19^. The abundance of scale-free dynamics in various natural phenomena raises the question of whether scale-free dynamics modulate the brain’s neuronal activity concerning consciousness and the neuronal activity’s alignment with environmental stimuli or inputs.

Scale-free physiological processes in time imply that no specific timescale or frequency drives the biological system^20^. Rather than showing dominant oscillations, events spread across a broadband 1/*f* pink noise power spectrum where power falls off as frequency increases. Notably, a resting-state functional MRI study^19^ demonstrated that scale-free brain dynamics, assessed via the power-law exponent (PLE) and corresponding to 1/*f* pink noise, collapsed under propofol-induced anesthesia. The PLE calculation follows a linear least-square regression of a power-law in the frequency domain’s log-power and log-frequency transformation. This resting-state study observed that pink noise in the BOLD’s infra-slow frequency band (0.01–0.1 Hz) under consciousness transformed into a flat white noise power spectrum in anesthesia where all frequencies approximately share the same power^19^. However, one unsolved question is how the loss of the brain’s intrinsic spontaneous activity’s (resting-state’s) pink noise during the loss of consciousness affects the brain’s task-related activity.

**Fig. 1:**
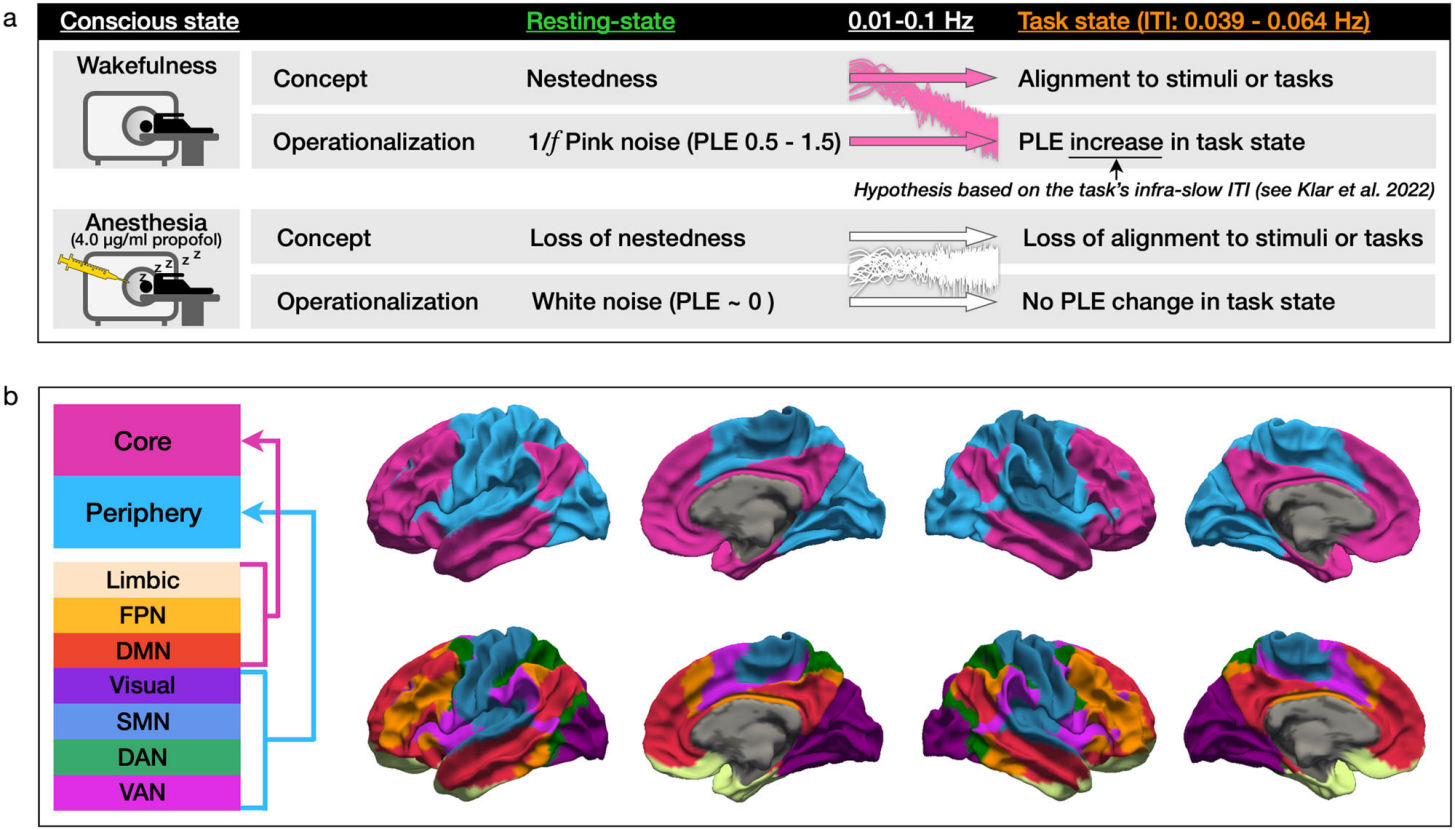
Overview of the investigated two mechanisms for consciousness in the core-periphery topography investigated in the fMRI infra-slow frequency band (0.01–0.1 Hz). **a** Two investigated mechanisms for consciousness, namely temporo-spatial nestedness and alignment, suggested by the Temporo-Spatial Theory of Consciousness (PLE power-law exponent, ITI inter-trial interval). **b** The assessed topography divided the human cerebral cortex into higher-order association core (colored in pink) and lower-order sensorimotor periphery (colored in blue) regions. Seven networks constitute the core-periphery topography. The core region comprises the limbic, frontoparietal, and default-mode networks, whereas the periphery region comprises the dorsal attention, ventral attention, somatomotor, and visual networks (see methods section for details). (DMN default-mode network, FPN frontoparietal network, DAN dorsal attention network, VAN ventral attention network, SMN somatomotor network.).

Neuroimaging studies investigated scale-free dynamics in conscious^14–18,20^ and unconscious states^19^. In contrast, it remains unknown if and to what extent the brain’s intrinsic scale-free dynamics undergo modulation by extrinsic stimuli, such as during task-related activity under the loss of consciousness. Regarding this question, the Temporo-Spatial Theory of Consciousness (TTC) suggests a mechanism described as temporo-spatial alignment^9,21^. Temporo-spatial alignment refers to the brain’s neuronal activity’s adaptation to the temporal and spatial structure of extrinsic stimuli or inputs during task-related activity. One paradigmatic empirical manifestation of temporo-spatial alignment is observable when the brain’s task-related activity follows the task’s temporal structure^18^. Temporo-spatial alignment, in turn, requires a sufficient degree of scale-free dynamics (or PLE level) of the brain’s ongoing spontaneous activity or resting-state, labeled temporo-spatial nestedness^21,22^. Addressing the empirical demonstration of the task-related temporo-spatial alignment for consciousness, including its close relationship with the resting-state’s temporo-spatial nestedness, sets the overall aim for our fMRI analysis in three levels of consciousness (awake, sedation, and anesthesia) displayed in Fig. 1.

Further neuroimaging studies highlighted the connection between the brain’s temporal dynamics and the cerebral cortex’s spatial topography under conscious wakefulness^6,23–26^. Paradigmatically, the brain’s intrinsic neuronal timescales, measured by the signal’s autocorrelation during the resting-state, followed a topographic division into higher-order transmodal association (core) and lower-order unimodal sensorimotor (periphery) regions in both fMRI^18,24–26^ and magnetoencephalography^23^. In this line of research, a recent fMRI analysis by our group^18^ provided evidence of how the resting-state’s scale-free dynamics assessed in the same core-periphery topography undergo modulation by the task’s temporal structure under conscious wakefulness, namely by the task’s inter-trial intervals. It remains an open question wherever the modulation of the brain’s intrinsic scale-free dynamics by the task’s temporal structure, reflecting an example of temporo-spatial alignment and nestedness, preserves in the core-periphery topography under the loss of consciousness. We addressed this question via three aims.

Aim one investigated wherever a systematic relationship holds between the brain’s intrinsic scale-free dynamics in the core-periphery topography, operationalized by the PLE^22,27^, and the level of consciousness across three conscious levels from awake, over sedation, to anesthesia. Based on a previous fMRI study^19^ and the TTC hypothesis of nestedness, we predicted that scale-free dynamics represent a potential predisposition or requirement for consciousness that, in turn, is lost under propofol-induced anesthesia.

Aim two tested the TTC alignment mechanism by investigating if and to what extent scale-free dynamics, measured by the PLE, show task-related modulations potentially based on the brain’s alignment with the task’s infra-slow temporal structure (0.039–0.064 Hz), across the three conscious states. Based on previous fMRI studies that showed task-related PLE changes^14^, and PLE increases in task designs comprising infra-slow inter-trial intervals^16^, we hypothesized a significant task-related PLE increase compared to the resting-state in the awake state. We hypothesized the loss of task-related PLE increases in anesthesia based on the brain’s substantially reduced reactivity to exteroceptive inputs or stimuli under deep anesthesia^28–31^ related to a loss of scale-free dynamics hypothesized for aim one. The second hypothesis (aim two), namely the relevance of scale-free dynamics and pink noise for the brain’s alignment with stimuli, is falsified if the PLE would still show significant task-related increases following the task’s temporal structure under the presence of potential white noise in anesthesia.

Aim three focused on the impact of the extrinsic input’s temporal structure. Aim three reached beyond aims one and two, which investigated the brain’s intrinsic noise colors and task-related activity but left unanswered the impact of the stimuli’s or task’s noise colors, such as paradigmatically white, pink, or brown noise. For that purpose, we employed the computational Stuart–Landau model^32–34^ to investigate to what degree different input strengths of white, pink, and brown noise affect a system of coupled oscillators near criticality. We hypothesized that power distributions of the extrinsic input, spanning from white noise to scale-free inputs by pink and brown noise, individually affect the model’s coupled oscillators. Support for this hypothesis can provide evidence that the brain’s alignment with the environment during conscious wakefulness does not solely depend on the brain’s intrinsic scale-free dynamics, i.e., temporo-spatial nestedness, but, furthermore, on the extrinsic dynamics of environmental inputs^35,36^.

In summary, the empirical results revealed a systematic relationship between scale-free dynamics and the level of consciousness in rest and task states. Power spectra showed increasing flattening towards white noise from sedation to anesthesia compared to the pink noise under conscious wakefulness. The computational results highlighted that the extrinsic input’s dynamics, besides the brain’s intrinsic scale-free dynamics, affected the model/brain-environment interaction. From the perspective of the TTC, our findings demonstrated the importance of two mechanisms for the level of consciousness, namely temporo-spatial nestedness and alignment. The second mechanism of alignment with the task’s temporal structure potentially depends on the kind of noise color, such as pink noise, regarding both (1) the brain’s intrinsic activity manifested by the first mechanism of nestedness and (2) the dynamical structure of extrinsic inputs further affecting the second mechanism of alignment.

## Results

### Scale-free dynamics decreased from wakefulness over sedation to anesthesia— loss of temporo-spatial nestedness

Aim one investigated the brain’s resting-state scale-free dynamics operationalized by the power-law exponent (PLE) in three states of consciousness: awake, sedation, and anesthesia/unconsciousness. We tested if the presence of the BOLD’s ~1/*f* pink noise potentially reflects a necessary neuronal predisposition of consciousness (NPC), thus probing the need for temporo-spatial nestedness as the first mechanism suggested by the TTC. The hypothesis is falsified when scale-free dynamics or pink noise remain intact and show topographic core-periphery differentiation in the BOLD’s infra-slow frequency band during the loss of consciousness in anesthesia.

**Fig. 2:**
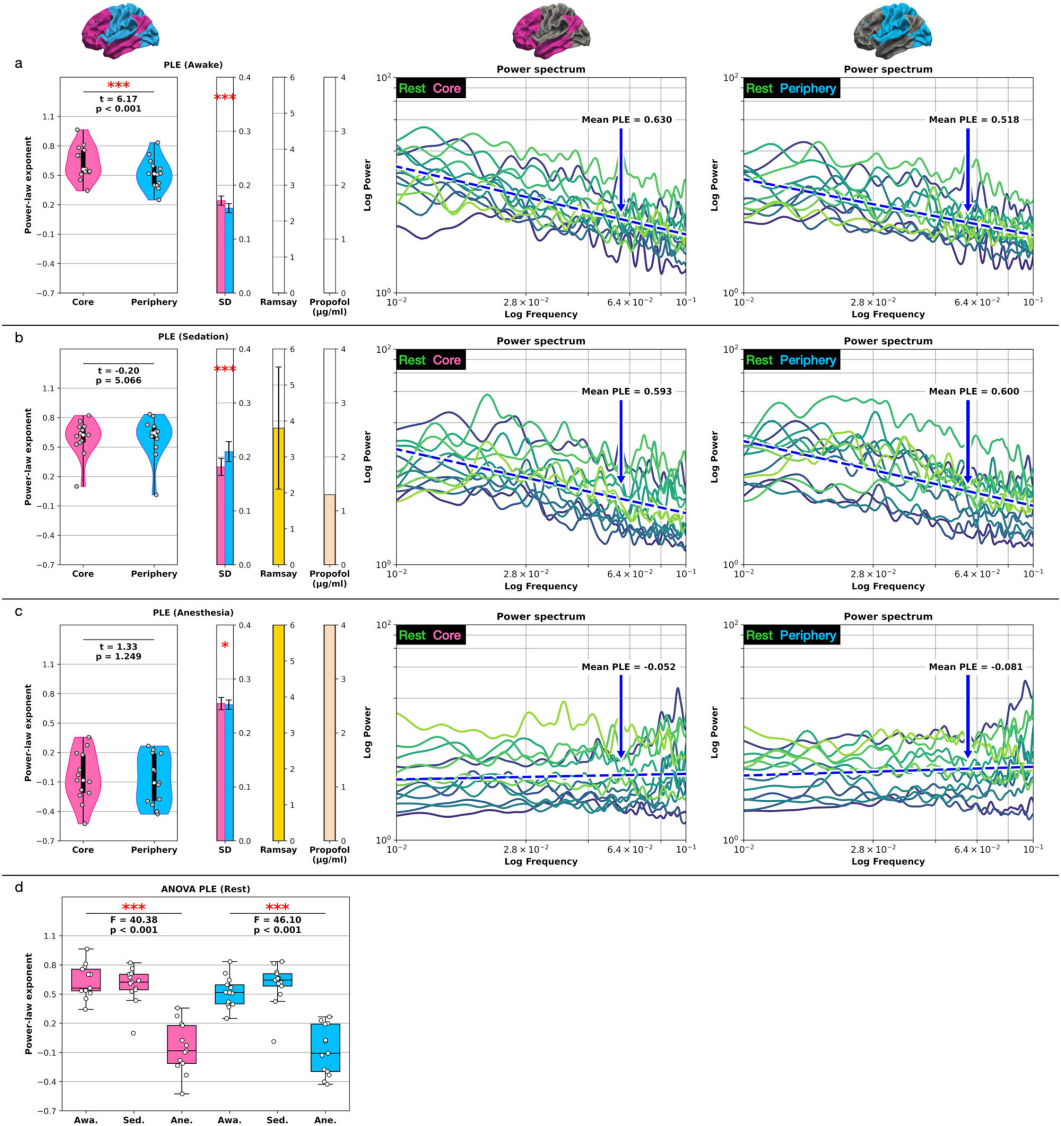
Resting-state inverse power-law distributions and PLE where each line represents one subject. The blue dashed line represents the mean linear least-square regression across all subjects. **a** The core-periphery comparison yielded significant PLE differences under conscious wakefulness. **b** The PLE significantly decreased and converged between the core and periphery regions in sedation. **c** The power spectra flattened to white noise under anesthesia/unconsciousness (0.01–0.1 Hz or 10^−2^ to 10^−1^ on the logarithmic scale). **d** One-way repeated measures ANOVA between the three states of consciousness. Error bars for the SD represent the standard error of the mean (SEM) based on 599 bootstrap samples and SD for the Ramsay score. Boxplot center line represents the median, boxes the interquartile range (IQR), and whiskers 1.5x IQR; *n* = 13 subjects. (PLE power-law exponent, SD standard deviation, Awa awake, Sed sedation, Ane anesthesia.).

Awake: We observed intact scale-free dynamics in the resting-state under conscious wakefulness, where the core PLE = 0.63 was significantly higher (*t* = 6.17, *p* < 0.001) than the periphery PLE = 0.518 displayed in Fig. 2a.

Sedation: The slope of the power-law distributions decreased under the sedative propofol concentration of 1.3 µg/ml in both core (PLE = 0.593) and periphery (PLE = 0.6) regions displayed in Fig. 2b. Additionally, the core-periphery division lost its statistical significance in sedation (*t* = −0.2, *p* = 5.066). The standard deviation (SD) increased from awake (core SD = 0.171, periphery SD = 0.157) to sedation (core SD = 0.181, periphery SD = 0.21). The relatively high SD was mirrored in the subjects’ behavioral response, where the Ramsay scores for consciousness likewise yielded substantial variability (mean/SD = 3.8/1.7).

Anesthesia: Subjects received a high propofol concentration of 4.0 µg/ml leading to unconsciousness in anesthesia which is also reflected in the highest Ramsay score of 6 for all subjects, indicating the loss of consciousness. Power spectra flattened to white noise for the complete BOLD’s infra-slow frequency band (0.01–0.1 Hz or 10^−2^ to 10^−1^ on the logarithmic scale) under anesthesia displayed in Fig. 2c. The horizontal power spectra caused a reduction of both the core PLE = −0.052 and periphery PLE = −0.081, indicating an uncorrelated random process. Like in the sedative state, the core-periphery division diminished in anesthesia (*t* = 1.33, *p* = 1.249).

ANOVA: In addition to paired *t*-tests between core and periphery, a one-way repeated measures ANOVA between the three states of consciousness was applied for core and periphery, respectively (see Fig. 2d and Supplementary Table 4). The three conscious states yielded a significant PLE difference in the core (*F* = 40.38, *p* < 0.001) and periphery region (*F* = 46.1, *p* < 0.001). Table 1 summarizes and Fig. 2 displays the resting-state PLE results in the core-periphery topography.

**Table 1:** Resting-state power-law exponent (PLE) core-periphery comparison.

Conscious state	Ramsay score	Run	Core	Periphery	*t* value	*p* value
Awake (Propofol 0 µg/ml)	1.0 ± 0	Rest	0.63 (0.171)	0.518 (0.157)	6.17	*p* < 0.001 ***
Sedation (Propofol 1.3 µg/ml)	3.8 ± 1.7	Rest	0.593 (0.181)	0.6 (0.21)	−0.2	5.066
Anesthesia (Propofol 4.0 µg/ml)	6.0 ± 0	Rest	−0.052 (0.254)	−0.081 (0.252)	1.33	1.249

Data represents region-based mean values across subjects including the standard deviation (SD) in brackets. The conscious state column includes the propofol concentrations. Statistics, Student’s paired *t*-test where *p* values are multiplied by six (Bonferroni correction); significance asterisks, *p* < 0.05 *, *p* < 0.01 **, *p* < 0.001 ***; *n* = 13 subjects.

### Scale-free dynamics no longer followed the task’s temporal structure in anesthesia —loss of temporo-spatial alignment

Aim two investigated the responsiveness of scale-free dynamics to the task’s infra-slow temporal structure (0.039–0.064 Hz) in the three conscious states, respectively. Aim two thus allowed testing the TTC hypothesis of a brain-environment alignment, namely temporo-spatial alignment, that, as we hypothesized, requires the presence of scale-free dynamics in the infra-slow band (0.01–0.1 Hz) during task states. We expected task PLE increases in the core-periphery topography under conscious wakefulness as previously observed in another dataset^18^, where the brain shifts power away from faster towards slower frequencies following the task’s infra-slow temporal structure (0.039–0.064 Hz).

Awake: In accordance with the task’s infra-slow temporal structure, the power-law distributions’ slope of both the core and periphery regions increased compared to the resting-state. The division between core (PLE = 0.697) and periphery (PLE = 0.627) diminished below statistical significance in the task state (*t* = 1.92, *p* = 0.475).

Sedation: The task PLE still increased under sedation compared to the resting-state in both core (PLE = 0.69) and periphery (PLE = 0.642). As observed in the awake task state, the core-periphery topography lacked statistical significance (*t* = 2.53, *p* = 0.157).

Anesthesia: As observed in the resting-state, power spectra flattened to white noise for the complete frequency band under anesthesia. The statistical difference between the core (PLE = −0.087) and periphery (PLE = −0.084) topography diminished further in the task state under anesthesia (*t* = −0.154, *p* = 5.282).

**Fig. 3:**
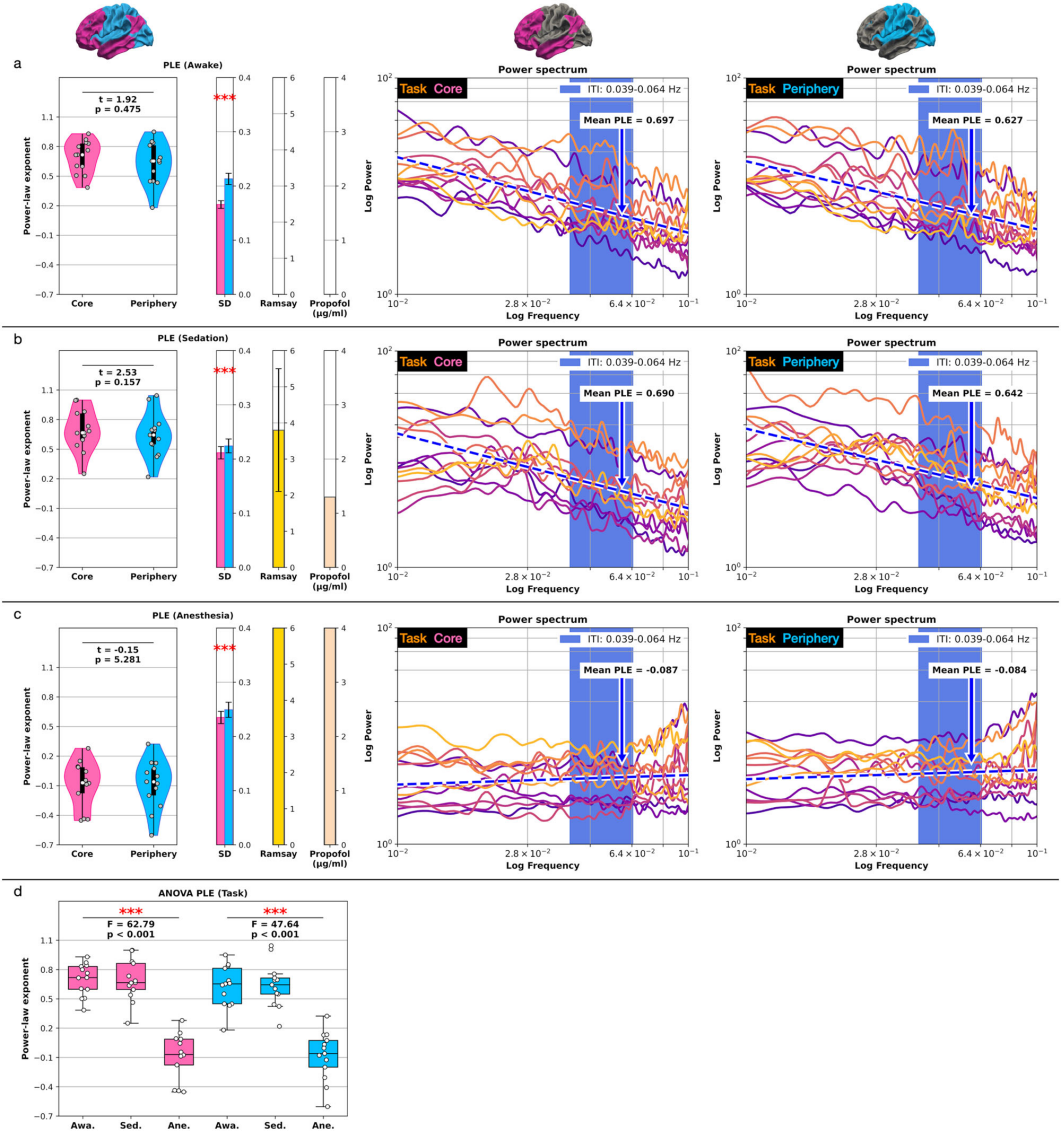
Task state inverse power-law distributions and PLE where each line represents one subject. The blue dashed line represents the mean linear least-square regression across all subjects. The blue shaded area represents the task’s frequency range (0.039–0.064 Hz). **a** The core-periphery comparison lacked significant PLE differences under conscious wakefulness. **b** The PLE significantly decreased and further converged between the core and periphery regions in sedation. **c** The power spectra flattened to white noise under anesthesia/unconsciousness (0.01–0.1 Hz or 10^−2^ to 10^−1^ on the logarithmic scale). **d** One-way repeated measures ANOVA between the three states of consciousness. Error bars for the SD represent the standard error of the mean (SEM) based on 599 bootstrap samples and SD for the Ramsay score. Boxplot center line represents the median, boxes the interquartile range (IQR), and whiskers 1.5x IQR; *n* = 13 subjects. (PLE power-law exponent, SD standard deviation, Awa awake, Sed sedation, Ane anesthesia).

ANOVA: The three conscious states yielded a significant PLE difference in the core (*F* = 62.79, *p* < 0.001) and periphery region (*F* = 47.64, *p* < 0.001). Table 2 summarizes and Fig. 3 displays the task PLE results in the core-periphery topography. Supplementary Table 3 summarizes the rest and task states ANOVA.

**Table 2:** Task state power-law exponent (PLE) core-periphery comparison.

Conscious state	Ramsay score	Run	Core	Periphery	*t* value	*p* value
Awake (Propofol 0 µg/ml)	1.0 ± 0	Task	0.697 (0.166)	0.627 (0.213)	1.92	0.475
Sedation (Propofol 1.3 µg/ml)	3.8 ± 1.7	Task	0.69 (0.212)	0.642 (0.224)	2.53	0.157
Anesthesia (Propofol 4.0 µg/ml)	6.0 ± 0	Task	−0.087 (0.235)	−0.084 (0.249)	−0.154	5.282

Data represents region-based mean values across subjects, including the standard deviation (SD) brackets. The conscious state column includes the propofol concentrations. Statistics, Student’s paired *t*-test where *p* values are multiplied by six (Bonferroni correction); significance asterisks, *p* < 0.05 *, *p* < 0.01 **, *p* < 0.001 ***; *n* = 13 subjects.

### PLE rest-task differences

Besides investigating the core-periphery regions individually in rest and task states, we calculated two further comparisons to assess the PLE change from rest-to-task states in all conscious levels (see the method section “Calculations of PLE rest-task differences” for details on the calculation).

**Fig. 4:**
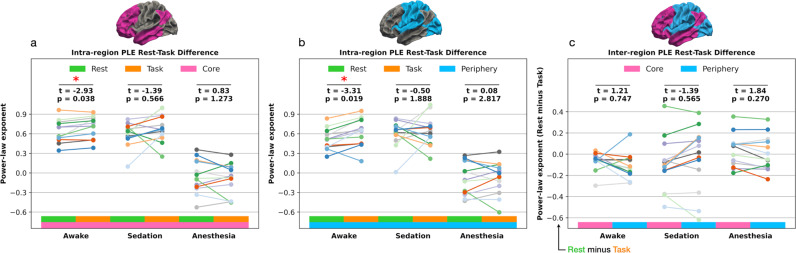
Intra-region and inter-region PLE calculations. The individual line plot colors represent single subjects (*n* = 13). **a** Intra-region rest-task difference for the core region statistically compared the rest-to-task PLE changes. **b** Same as in (**a**) but for the periphery region. **c** Inter-region rest-task difference calculated the rest-to-task PLE changes within the core and periphery region, respectively.

1. Intra-region rest-task difference: We compared the rest against task PLE within both core and periphery. This difference shows the statistical PLE increase within each region, i.e., the statistical significance of the core and periphery rest-to-task PLE increases. Awake: Scale-free dynamics in the task state under conscious wakefulness significantly increased compared to the awake resting-state for both core (*t* = −2.93, *p* = 0.038) and periphery (*t* = −3.31, *p* = 0.019) regions. Sedation: The task PLE still increased under sedation compared to the resting-state in both the core and periphery regions. However, the rest-to-task PLE increase was below statistical significance for both the core (*t* = −1.39, *p* = 0.566) and periphery (*t* = −0.5, *p* = 1.888). Anesthesia: Compared to the resting-state, the task state PLE no longer increased in both core (*t* = −0.83, *p* = 1.273) and periphery (*t* = 0.08, *p* = 2.817). In summary, diminished scale-free dynamics observed under 1.3 µg/ml of propofol in sedation led to non-significant PLE changes in task states. The same observation holds for higher propofol concentrations of 4.0 µg/ml in anesthesia.
2. Inter-region rest-task difference: We subtracted task from rest PLE levels individually for the core and periphery regions. This calculation highlights the absolute PLE increases from rest-to-task states. Subsequently, we compared the rest-task difference results between the core and periphery regions, testing whether the rest-task difference was higher in the core or periphery region. Awake: The rest minus task PLE lacked statistical significance (*t* = 1.21, *p* = 0.747) between the core and periphery regions. Sedation: The rest minus task PLE difference between the core and periphery regions turned out non-significant (*t* = −1.39, *p* = 0.565). Anesthesia: Like in awake and sedation, the rest minus task PLE difference between the core and periphery regions was far from statistical significance (*t* = 1.84, *p* = 0.27). In summary, the rest minus task PLE difference between core and periphery lacked statistical significance in all conscious states. Figure 4 and Table 3 display the rest-task calculations.

**Table 3:** Power-law exponent (PLE) rest-task differences.

Conscious state	Region	Rest	Task	*t* value	*p* value
Awake (Propofol 0 µg/ml; Ramsay 1.0 ± 0)	Core	0.63 (0.171)	0.697 (0.166)	2.93	0.038 *
	Periphery	0.518 (0.157)	0.627 (0.213)	3.31	0.019 *
Sedation (Propofol 1.3 µg/ml; Ramsay 3.8 ± 1.7)	Core	0.593 (0.181)	0.69 (0.212)	1.39	0.566
	Periphery	0.6 (0.21)	0.642 (0.224)	0.5	1.888
Anesthesia (Propofol 4.0 µg/ml; Ramsay 6.0 ± 0)	Core	−0.052 (0.254)	−0.087 (0.235)	−0.83	1.273
	Periphery	−0.081 (0.252)	−0.084 (0.249)	−0.08	2.812

Data represents region-based mean values across subjects, including the standard deviation (SD) in brackets. Statistics, Student’s paired *t*-test; significance asterisks, *p* < 0.05 *, *p* < 0.01 **, *p* < 0.001 ***; *n* = 13 subjects.

### Seven networks PLE analysis

In addition to PLE analyses for the core-periphery topography, we analyzed the PLE in all seven networks that constituted the core-periphery topography. First, the seven network PLE analysis allowed validation that PLE differences between core and periphery regions also occur on the single network level. Second, we aimed to control that different PLE levels across the three conscious states are manifest on the single network level. Third, we computed a hierarchy via a linear least-square regression across the seven networks’ PLE levels. We analyzed this hierarchy for the three conscious levels in rest and task states, respectively. This analysis allowed us to highlight a potentially diminishing hierarchy in sedation or its absence in anesthesia compared to conscious wakefulness.

Awake: The resting-state PLE level increased from peripheral, e.g., limbic and SMN, to core networks, e.g., FPN and DMN. Additionally, the mean PLE increased in all networks from the rest to the task state.

Sedation: Following the core-periphery results, the mean PLE remained stable across most of the seven networks under sedative propofol levels, matching the seven networks PLE levels observed under conscious wakefulness. On average, as observed for the core-periphery topography, the PLE still increased in the task state, albeit to a lesser extent than under conscious wakefulness.

Anesthesia: The mean PLE levels in all networks drastically diminished under the high propofol dosage and the loss of consciousness in anesthesia, reaching levels of ~ 0 as observed for the core-periphery topography. The rest vs. task PLE difference remained non-significant results for all seven networks (*p* ≥ 3.165).

**Fig. 5:**
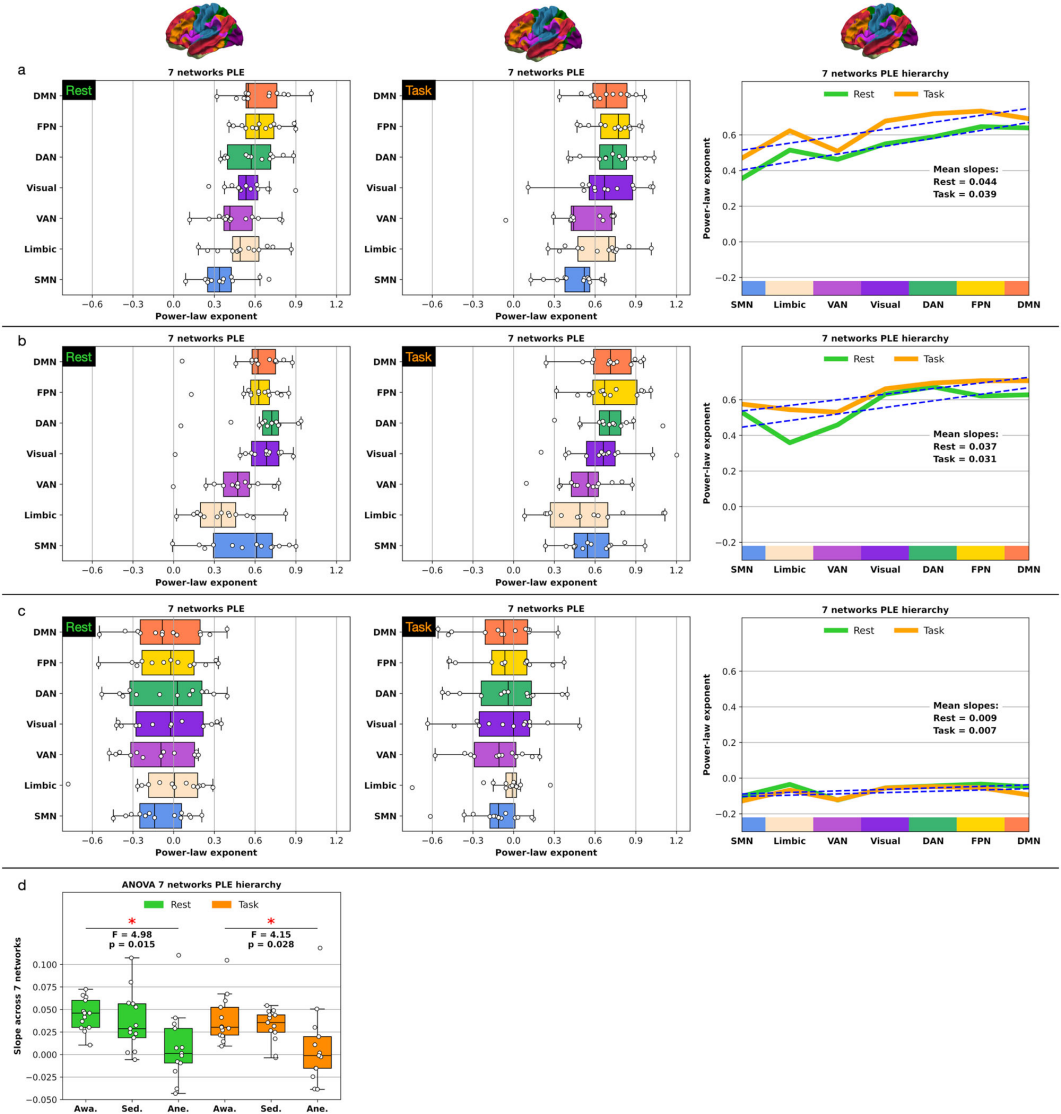
Seven networks PLE levels and PLE hierarchy across the seven networks. The PLE hierarchies (right) display the mean slope and regression across all subjects. **a** PLE in rest and task states under consciousness. **b** PLE in rest and task states under sedation. **c** PLE in rest and task states under anesthesia. **d** One-way repeated measures ANOVA between the three states of consciousness in rest and task. The blue dashed lines in the hierarchies (right) represent the least-square linear regression across the seven networks. Boxplot center line represents the median, boxes the interquartile range (IQR), and whiskers 1.5x IQR; *n* = 13 subjects. (DMN default-mode network, FPN frontoparietal network, DAN dorsal attention network, VAN ventral attention network, SMN somatomotor network.).

In summary, the seven network hierarchy showed the highest mean slopes under conscious wakefulness for rest (slope = 0.044) and task (slope = 0.039) states. As anticipated, the mean slopes decreased under sedation for rest (slope = 0.037) and task (slope = 0.031) states. Finally, the hierarchy dissolved into horizontal planes under anesthesia for rest (slope = 0.009) and task (slope = 0.007) states. Overall, the seven network rest vs. task PLE results mirrored the previous observations of the core-periphery topography across all conscious states. The results are shown in Fig. 5 and summarized in Supplementary Tables 5, 6.

### Computational modeling: impact of the extrinsic input’s different noise colors

**Fig. 6:**
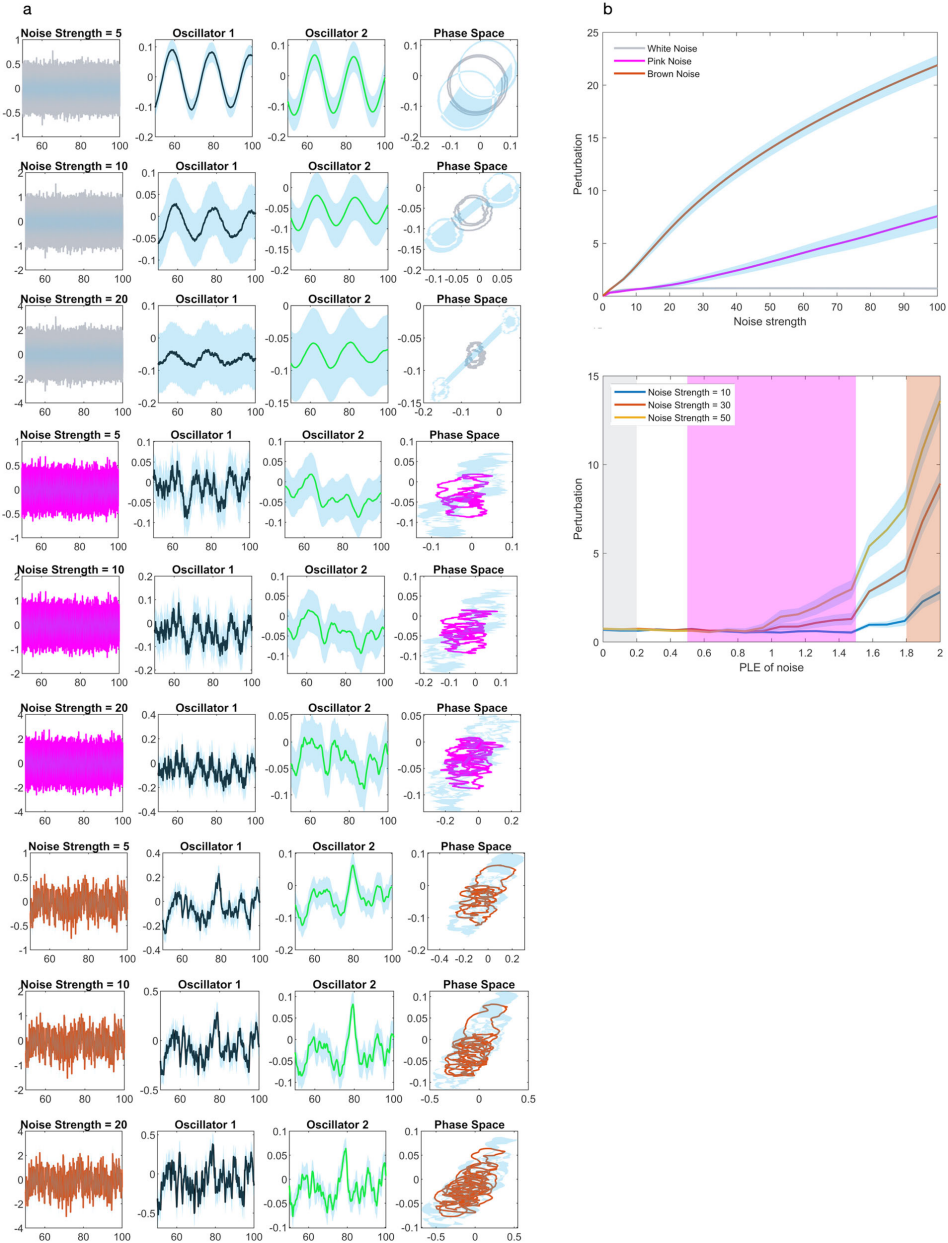
The effect of different inputs on the Stuart–Landau model of two coupled oscillators. **a** We provided the model with three different noise colors of white, pink, and brown noise, including three logarithmically increasing noise strengths from 0.1 to 100 in 20 simulations for each noise color. As can be seen in the phase diagrams, inputs with a scale-free temporal structure can easily perturb a system even with low input strength, whereas white noise inputs require very high input strength to affect the system. Lines show the mean across trials, and shadings show the standard deviations. **b** The effect of the PLE of noise and noise strength on perturbation which was defined as the absolute value of the difference in the area of the circle in the phase space between perturbed and not perturbed states. Lines show the mean, and the shadings show the standard error.

The presented empirical findings showed that the loss of the brain’s scale-free dynamics, namely temporo-spatial nestedness, from pink noise under conscious wakefulness to white noise under unconsciousness, corresponded to degrees of the brain’s task-related activity or reactivity to extrinsic inputs, namely temporo-spatial alignment. While these results demonstrated a role of the brain’s intrinsic scale-free dynamics, they did not probe the impact of the extrinsic inputs’ dynamics on the brain’s temporo-spatial alignment. To assess this question, we employed the computational Stuart–Landau coupled oscillator model. We simulated inputs in three strength levels and noise colors, spanning from white over pink to brown noise, assessing their impact on a coupled oscillator system near criticality shown in Fig. 6a, b.

Even with low noise strength, the brown noise input, indexing a high degree of scale-free dynamics, already desynchronized the system. On the other hand, white noise, indexing the loss of scale-free dynamics or temporo-spatial nestedness, required very high input strengths to induce changes in the model’s degree of synchronization. Pink noise inputs yielded desynchronization effects in the model’s activity that stood between the extremes of those observed during white and brown noise inputs. In sum, these modeling results demonstrated the importance of the extrinsic input dynamics for the brain’s task-related responses, namely temporo-spatial alignment. Based on the model’s results together with the empirical findings, we assume that the brain’s task-related activity, namely the degree of temporo-spatial alignment, depends upon the degree of scale-free dynamics reflected in noise color (white, pink, or brown noise) of both the brain’s intrinsic activity and the extrinsic input’s dynamics.

### PLE control and replication analyses

PLE control analysis I: the distinction between fractal and oscillatory components (IRASA): The IRASA method^37^ was applied to separate oscillatory and fractal components of the power spectrum, previously successfully measured in electroencephalography (EEG) and magnetoencephalography (MEG) recordings^20,37,38^. Supplementary Fig. 1 displays the comparison between the conventionally computed PLE presented above, including both fractal and oscillatory components in the power spectrum, and the IRASA method obtained fractal-based PLE levels (exclusion of oscillatory components) in rest and task states. The comparison between both analysis methods yielded no significant differences. The results indicate that our PLE results were not driven by oscillatory components, but reflected a genuine change in the power spectras’ fractal component, that is, in scale-free dynamics.

PLE control analysis II: Comparison with surrogate data: We tested wherever the measured power spectra were scale-free by comparing the goodness of fit of the power-law to the PSDs of real data and simulated fractional Gaussian noise (fGn)^14,39–41^. The core and periphery regions had *ρ*-values, that is, the fraction of synthetic time-series that had a worse fit than real data, exceeded 0.05, except for the awake task in the periphery region (0.021) and anesthesia task in the core region (0.045). The results provided further evidence for genuine scale-free dynamics in the awake state and are displayed in supplementary Table 1.

Replication analyses results: We computed two additional control analyses to back up our PLE observations. In the following, we only present a brief overview of the results.

1. The computed task time windows (volumes 90–325 and 329–564), matched to the resting-state length (236 volumes), showed the same rest-to-task PLE increase in the awake and sedative states and the loss of task-related PLE changes in anesthesia that we observed for the full-length task runs. Additionally, mirroring the full-length task run results, the PLE levels between the core and periphery regions converged below statistical significance in both task time windows for all conscious states. Supplementary Figs. 2, 3 and Supplementary Table 2 display the results.
2. The mean frequency (MF) analysis followed the PLE results in the awake state’s core vs. periphery comparison by showing a significant difference (*t* = −4.64, *p* = 0.003) between both regions in the resting-state that disappeared in the task state (*t* = −1.75, *p* = 0.636). The MF results in sedation also followed the PLE findings, lacking a significant difference between the core and periphery regions in rest (*t* = 1.09, *p* = 1.772) and task (*t* = −0.02, *p* = 5.915) states. Finally, the anesthetic state showed no significant difference between the core and periphery regions in the resting-state (*t* = −2.06, *p* = 0.373) and in the task (*t* = −1.69, *p* = 0.702) state. Consequently, MF patterns for the resting-state and task state matched the PLE results. Supplementary Figs. 4, 5 and Supplementary Table 3 display the results.

## Discussion

The empirical fMRI and computational analyses yielded three main results. (1) The brain’s resting-state comprised different degrees of scale-free dynamics ranging from pink noise under conscious wakefulness to the loss of scale-free dynamics and white noise in anesthesia. (2) The brain’s scale-free dynamics showed significant task-related PLE increases potentially related to the task’s temporal structure only under conscious wakefulness^18^. Conversely, these task-related PLE changes diminished in sedation and vanished under the loss of consciousness in anesthesia. (3) The external input’s dynamics of white, pink, or brown noise, representing stimuli, yielded different degrees of task-related changes demonstrated by the computational model. Findings one to three provide evidence for the suggested mechanisms of temporo-spatial nestedness and alignment, including their interactive relationship, suggested by the Temporo-Spatial Theory of Consciousness (TTC).

The Temporo-Spatial Theory of Consciousness (TTC) hypothesizes that scale-free brain dynamics, namely temporo-spatial nestedness, are potentially a necessary albeit non-sufficient neuronal predisposition of consciousness^21^. More precisely, the hypothesis of temporo-spatial nestedness suggests that a scaling relationship between frequency and power, where faster but less powerful frequencies are nested into slower yet more powerful frequencies, is needed for consciousness.

Following the first TTC mechanism, our findings demonstrated a systematic relationship between scale-free dynamics of the brain’s ongoing spontaneous activity, assessed via the resting-state, and the state or level of consciousness. Inverse power-law distributions showing pink noise (PLE = 0.5 to 1.5) were present under conscious wakefulness across the core-periphery topography. The intensity of pink noise diminished under sedation, and pink noise completely vanished in exchange for white noise for the complete power spectrum under anesthesia (0.01–0.1 Hz or 10^−2^ to 10^−1^ on the logarithmic scale). The presence of pink noise suggests that a mixture between variability and regularity that characterizes pink noise with its ubiquitous presence in both the body^3,4,42^ and the brain’s physiology^43,44^ could present a predisposition for consciousness. Conversely, white noise comprises high variability and lack of regularity which, as observed in the BOLD’s infra-slow frequency band during anesthesia, dominated during the loss of consciousness.

Interestingly, under sedation and anesthesia the subjects showed a higher standard deviation, compared to the awake state, for the PLE and MF in resting-state and task and a high standard deviation in the Ramsay scores (mean/SD = 3.8/1.7). Different subjects may exhibit various degrees of reactivity to the low-level propofol concentration (1.3 µg/ml) used for sedation. Propofol is a c-aminobutyric acid (GABA) receptor agonist that exerts its hypnotic effect through the potentiation of the inhibitory GABA neurotransmitter^45^. Propofol in low concentration potentiates inward chloride currents, while at higher concentrations, propofol directly causes channel opening^46^. Low propofol concentrations potentially best revealed higher degrees of inter-subject variability of the brain’s reactivity under sedative and anesthetic propofol concentrations. This well-known inter-subject variability to anesthetic drugs is further rooted in gene polymorphisms. Polymorphisms then influence the pharmacokinetics and pharmacodynamics of propofol^47–49^.

One reason why various PLE levels across the cerebral cortex’s topography are relevant for consciousness might lie in what we conceptualize as (1) temporal nestedness and (2) functional-topographic nestedness, which both converge as a potential requirement for consciousness. The nested structure manifests itself in the brain’s ongoing spontaneous activity and the alignment of the former to environmental inputs observed in power spectra, namely temporal nestedness, and additionally in a quasi-nested functional topography observed across brain regions and networks. More precisely and regarding the core-periphery topography, we observed lower PLE levels in the periphery and higher in the core (see Figs. 2 and 3), while in the seven networks, the lowest PLE levels accordingly occurred in the limbic and somatomotor and highest in the DMN and FPN networks under conscious wakefulness (see Fig. 5). Conversely, the PLE levels not only reduced to approximately zero under the loss of consciousness in anesthesia, reflecting a loss of temporal nestedness, but the core-periphery difference and the seven network hierarchies vanished as well, reflecting an additional loss of functional-topographic nestedness. We suggest that the cerebral cortex requires a repertoire of scale-free dynamics, where the dynamics of lower-order unimodal networks are functionally (virtually) nested in the dynamics of transmodal association cortices or networks. The loss of the convergence between temporal and functional-topographic nestedness might result in the loss of integration into a functional temporal-spatial space for brain dynamics, whose disruption leads to the loss of consciousness.

In addition to scale-free dynamics, namely temporo-spatial nestedness, the TTC suggests that a temporo-spatial alignment of the brain’s neuronal activity to its respective environmental context represents another mechanism for consciousness^21^. Consciousness requires the brain’s intrinsic activity to align with the extrinsic interoceptive bodily and exteroceptive environmental inputs. This interaction may prove central for the level or state of consciousness while yielding what we empirically observe as stimulus- or task-evoked activity on the neuronal level.

The core-periphery topography showed a significant PLE increase that followed the task’s temporal structure only under conscious wakefulness (core: *t* = 2.93, *p* = 0.038; periphery: *t* = 3.31, *p* = 0.019). Already in sedation, the rest-to-task PLE increase diminished below statistical significance under a low-level propofol concentration of 1.3 µg/ml, corresponding to an initial departure from scale-free dynamics. Propofol-induced unconsciousness in anesthesia (4.0 µg/ml) resulted in white noise power spectra, i.e., a loss of scale-free dynamics where rest-to-task PLE changes vanished. These findings hint towards a systematic relationship between scale-free dynamics of the brain’s ongoing spontaneous activity and the former’s reactivity to environmental stimuli, as operationalized by task states across the three conscious levels. Hence, the brain’s intrinsic 1/*f* pink noise seemed important for the brain’s PLE’s reactivity and its neuronal following of the task’s infra-slow temporal structure. A hypothesis regarding the functional implication of the brain’s scale-free dynamics is that the former may require the complexity inherent to pink noise, where variability and regularity are intertwined, to entrain or encode environmental stimuli that, in turn, are often pink-colored as well^1,2,6,50–55^.

Simultaneously and in addition to the brain’s intrinsic and ongoing spontaneous activity, the extrinsic input’s dynamics during the task state potentially likewise carry importance for consciousness, supported by our computational findings. The Stuart–Landau model further demonstrated that pink and brown noise (scale-free) inputs easily perturbed the system even at low input strengths. Conversely, white noise and its lack of scaling required very high input strengths to affect the system.

Yu, Romero, and Lee^35^ demonstrated that pink noise stimuli are best transmitted by sensory neurons in the visual cortex (V1) compared to stimuli comprising either extensive long-range correlations, i.e., brown noise (1/${f}^{2}$), or no temporal correlation, i.e., white noise (1/${f}^{0}$). Qu and colleagues^36^ showed that neurons exhibited the highest firing rate, spike timing reliability, and encoding intensity to pink noise stimuli. In the same line, Soma and colleagues^56^ showed that pink noise better sensitizes the baroreflex centers in the brain compared to white noise, implying a better information transfer for pink noise compared to brown or white noise. The empirical results of our fMRI analysis extend these findings. We demonstrated that it is not only the extrinsic stimuli’s noise color that determines the environment’s impact on brain activity shown by our application of the Stuart–Landau model.

**Fig. 7:**
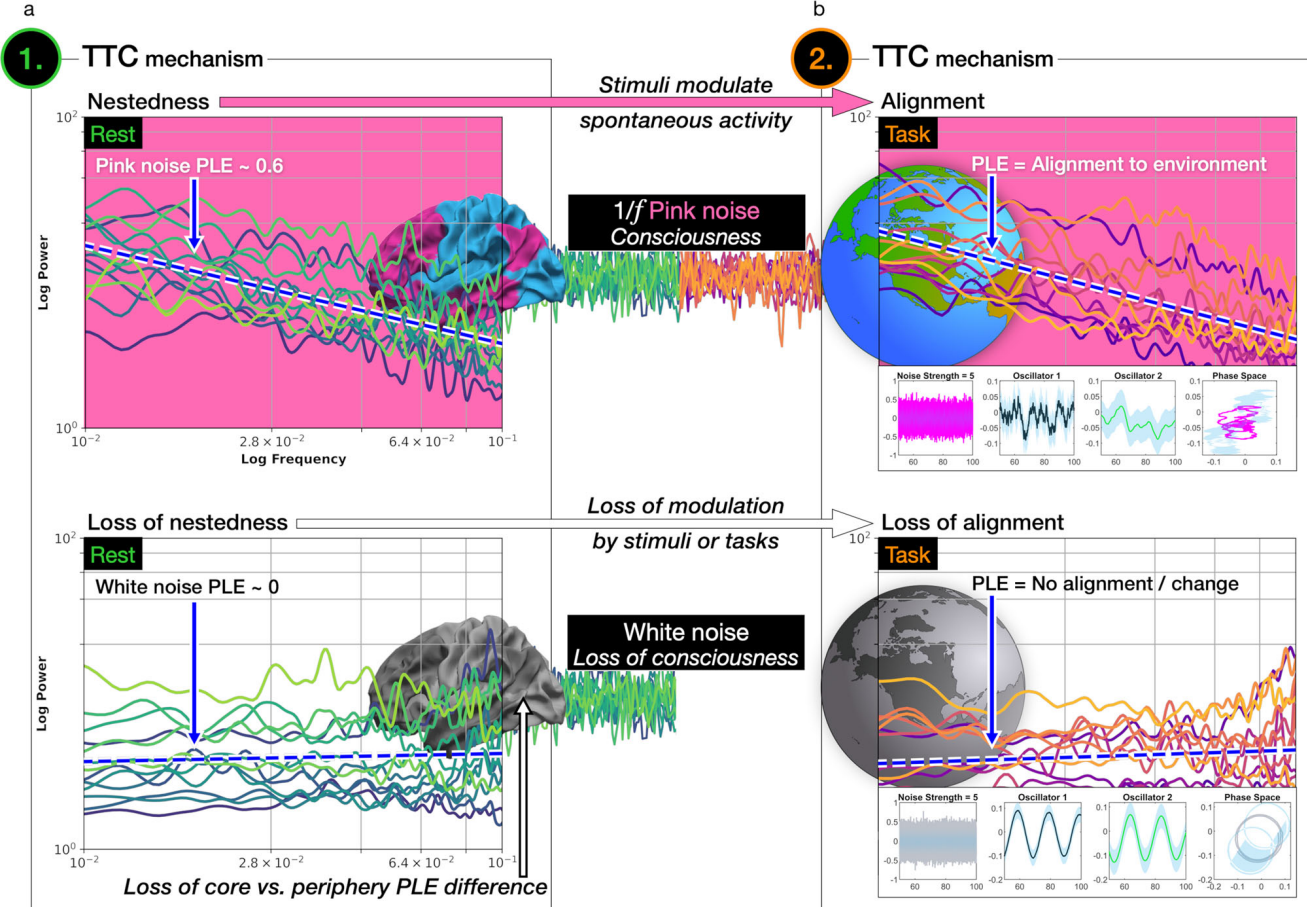
Two suggested mechanisms for consciousness by the Temporo-Spatial Theory of Consciousness. **a** Temporo-spatial nestedness. Scale-free dynamics in the vicinity of pink noise (PLE = −1) were observed under conscious wakefulness in the resting-state, whereas a partial flattening of power to white noise under propofol-induced anesthesia. **b** Temporo-spatial alignment. A significant rest-to-task PLE increase in response to the infra-slow inter-trial interval (0.039–0.064 Hz) only occurred under pink noise in the brain’s ongoing activity. Conversely, under the loss scale-free dynamics in anesthesia, the power distribution’s adaption or alignment in response to the task vanished.

Our analysis suggests the necessity of considering both the brain’s intrinsic and the environment’s extrinsic noise colors to understand the brain-environment interaction, including its relevance for consciousness. The idea that a system comprising complex dynamics, such as pink noise in the human brain, responds best to other systems that approximately share the same dynamics describes complexity matching^43,57,58^. Many empirical findings shed light on complexity matching between systems, such as the human body, and environmental stimuli^57,59–62^. Accordingly, in our computational model, scale-free pink and brown noise dynamics elicited far superior impacts even when using low input strengths on the two coupled oscillators compared to white noise with its lack of scaling. We, therefore, suggest that the brain’s scale-free dynamics potentially nest or integrate themselves within environmental dynamics to establish task-related PLE changes following the task’s temporal structure. Hence, the two mechanisms of temporo-spatial nestedness and alignment are potentially required to constitute the awake state’s high level of consciousness, including adaption to environmental stimuli or tasks displayed in Fig. 7.

### Limitations

Due to our empirical fMRI dataset constraints, testing different noise color inputs was empirically impossible. Therefore, we applied a computational model to simulate and test the impact of three noise colors and different strengths on synchronization-based oscillators. The modeling of the brain as coupled oscillators has been used extensively in the literature, thus providing validity to the Stuart–Landau model^61–69^. We confined the model to two oscillators for tractability of the results regarding the perturbation of the limit cycle. The simulation results confirmed our hypothesis by demonstrating that scale-free dynamics of pink and brown noise inputs yielded the highest degree of activity change, whereas white noise inputs lacked a high impact on the model’s coupled oscillators. Future analyses require systematic designs that allow testing the brain-environment interaction based on real instead of simulated stimuli by recording the stimulis’ time-series allowing the analysis in conjunction with brain dynamics from neuroimaging.

The recorded fMRI BOLD signal captures the hemodynamic deoxy-to-oxy rate inside the infra-slow frequency band (0.01–0.1 Hz). Comparing scale-free dynamics recorded via fMRI BOLD with electrophysiological signals by electroencephalography (EEG) and magnetoencephalography (MEG) is problematic. Paradigmatically and in opposition to fMRI findings, several EEG studies observed PLE increases instead of decreases under the loss of consciousness in anesthesia^31,70–72^. One possible theoretical inference for fMRI BOLD and electrophysiological recordings is that scale-free dynamics, measured by the PLE, must operate within a balanced scaling range: substantially changed PLE levels, either too high or low, may lead to the loss of consciousness. It remains unknown why pink noise or inverse power-law distributions of the BOLD signal corresponded with consciousness in this and other fMRI analyses^19^ since there is not one possible mechanistic explanation for scale-free dynamics or power-law scaling of physiological time-series recordings, but likely many^3^. We are still far from developing models grounded in a mechanistic and systematic understanding of why scale-free dynamics of hemodynamic and electrophysiological brain recordings are probably necessary for consciousness.

Furthermore, we interpreted our findings in light of the Temporo-Spatial Theory of Consciousness (TTC) while neglecting other neuroscientific theories of consciousness that potentially draw different theoretical inferences for the obtained results. A feature of the TTC is that it integrates the brain’s spontaneous activity with task-evoked activity^73^, coined rest-stimulus or rest-task interaction^21,22^. The observations in this analysis demonstrated that the spontaneous activity’s scale-free dynamics, namely temporo-spatial nestedness, modulated the degree of task-evoked activity, namely temporo-spatial alignment. The interaction between rest and task states represents a requirement for consciousness suggested by the TTC. Neuroscientific theories of consciousness often focus on either the resting-state^74,75^ or task-evoked activity^76,77^. (See refs. ^73,78–80^ for a detailed comparison between neuroscientific theories of consciousness.) This rest-task integration distinguishes the TTC from other neuroscientific theories of consciousness, such as paradigmatically the Integrated Information Theory (IIT)^77^ and Global Neuronal Workspace Theory (GNWT)^76^, where the focus is primarily on task-evoked activity^81^.

## Methods

### Subjects

For the fMRI analysis, we re-used data from 20 right-handed adults (male/female: 8/12; age 32–64 years) from a previous clinical study^82^. Subjects were undergoing an elective transsphenoidal approach for a resection of a pituitary microadenoma. The pituitary microadenomas diagnoses relied on their size (<10 mm in diameter without growing out of the sella). The diagnosis included radiological examinations and plasma endocrinal parameters. The subjects received physical status grades I or II (American Society of Anesthesiologists) with no history of craniotomy, cerebral neuropathy, vital organ dysfunction, or administration of neuropsychiatric drugs. Seven out of twenty subjects had to be excluded from the fMRI analysis due to excessive head motion during scanning, leaving thirteen subjects for our analysis. See the section “Functional MRI data preprocessing” for exclusion criteria.

### Anesthesia protocol

The original study^82^ includes the complete anesthesia protocol. We only present the propofol concentrations for the reduced conscious level under sedation and the loss of consciousness in anesthesia. A target-controlled infusion (TCI) pump allowed a constant effect-site concentration of propofol, estimated by the pharmacokinetic model^83^. The stable effect-site concentration of TCI propofol was 1.3 µg/ml for sedation and 4.0 µg/ml for anesthesia, where the 4.0 µg/ml concentration reliably induces the loss of consciousness^84,85^. Additionally, the conscious state was evaluated throughout the study using the Ramsay scale^83^. The subjects were asked to strongly squeeze the investigator’s hand. The subject was rated fully awake if the response to verbal command (“squeeze strongly my hand!”) was strong (Ramsay 1-2), in mild sedation if the response to the verbal command was clear but slow (Ramsay 3-4), and in anesthesia, if there was no response to the verbal command (Ramsay 5-6). The sedative state (1.3 µg/ml) yielded Ramsay scores (mean/SD) of 3.8 ± 1.7 and in the anesthetic state (4.0 µg/ml), all subjects showed the maximum Ramsay score of 6.0 ± 0. The Ramsay scores in anesthesia suggest that all subjects lost consciousness.

### Data acquisition

A Siemens 3T Magnetom scanner with a standard head coil acquired scans of the whole brain via gradient-echo echo-planar imaging (EPI) (33 slices, slice thickness = 5 mm, time repetition = 2000 ms, time echo = 30 ms, flip angle = 90°, field of view = 210 mm^2^, image matrix = 64 mm^2^). The original study^35^ acquired resting-state and task runs for the three conscious states of awake (wakefulness), sedation, and propofol-induced anesthesia. The subjects’ heads were fixed in the scan frame and padded with spongy cushions to reduce head motion. The eyes closed resting-state runs each comprised 236 volumes (7:52 min), and the task runs comprised 565 volumes (18:50 min). The study included high-resolution T1-weighted anatomical images.

### Task design

The dataset’s task paradigm^82^ offered a temporally sparse event-related design comprising 60 trials. These trials included 30 own and 30 other (an unknown person’s name) names delivered in a pseudo-random order. These names were recorded by a familiar voice from one of the subjects’ family members with an audio clip lasting for 0.5 s. See refs. ^86,87^ for details of this previously applied paradigm. The unknown names were individually matched to each patient’s name by gender and number of syllables. Inter-trial intervals (ITI) ranged from 15.5 to 25.5 s (frequency range = 0.039–0.064 Hz), jittered in 2-s steps. The long ITI provides sufficient time for the BOLD undershoot and the return to the baseline level of ongoing brain activity^88–90^. The long ITI avoids potential nonlinearities caused by overlapping hemodynamic responses between succeeding trials (see Fig. 1 in ref. ^22^). Stimuli were programmed using E-Prime (Psychology Software Tools, Pittsburgh, PA) and delivered via an audiovisual stimulus presentation system designed for an MRI environment. The headphone’s volume was adjusted to the comfort level of each subject. Subjects were required to pay attention and listen to the names without behavioral response or judgment.

### Functional MRI data preprocessing

Preprocessing was performed using AFNI (https://afni.nimh.nih.gov)^91^ applying the following steps: (1) removing the first four volumes of each functional run; (2) despiking and slice timing correction; (3) co-registration with high-resolution T1-weighted anatomical images; (4) nonlinear spatial normalization of the anatomical scans into MNI152 2009c space and subsequent nonlinear functional to anatomical alignment (normalization); (5) functional resampling to 3 × 3 × 3 mm^3^ voxels; (6) regression of linear and nonlinear drift (equivalent to high-pass filtering of 0.0067 Hz) plus averaging of eroded white matter (WM) and cerebrospinal fluid (CSF) signals to reduce non-neuronal noise^92^; (7) spatial smoothing using an 8 mm full-width at half-maximum isotropic Gaussian kernel. Volumes with head motion displacements >0.35 mm or rotation >3.5° were censored in both rest and task runs. We excluded subjects exhibiting more than 10% censored volumes from further data analysis.

### Statistics and reproducibility

We applied paired *t*-tests between the core and periphery regions as well as between rest and task states where, in both cases, we specified the alternative hypothesis as two-sided. Additionally, we applied one-way repeated measures ANOVAs between the three states of consciousness for rest and task states, respectively. The *t*-Test and ANOVA share the assumptions of parametric statistics, meaning that both tests make certain assumptions about the population’s distribution from which the sample was drawn (see, for instance ref. ^93^). We tested two assumptions of parametric tests. First, we controlled the data’s approximate normality within each group, i.e., in core and periphery for rest and task states, via the Shapiro–Wilk test. Second, we tested the assumption of the data’s approximate homogeneity of variance via the Levene test. Results: All samples passed the Shapiro–Wilk and Levene tests, meaning that the samples’ significance always showed *p* > 0.05. Consequently, we did not reject the null hypotheses of normality and homogeneity.

We used the Bonferroni correction to counterbalance the problem of multiple comparisons encountered in our analyses^94,95^. The Bonferroni correction counterbalances the multiple comparisons problem, namely the increased chance of obtaining false-positive comparisons. Instead of dividing the p-thresholds by the number of comparisons, paradigmatically *p* < 0.05/*n* where *n* is the number of comparisons, we multiplied the observed *p* values by *n*. The multiplication preserves the commonly used *p* thresholds for statistical significance of *p* < 0.05, *p* < 0.01, and *p* < 0.001 instead of lowering the *p* thresholds. Our analyses included different numbers of *t*-tests across the three conscious states, including their rest and task runs. We accordingly applied multiplication factors depending on the number of comparisons as follows.

1. The comparison of the core vs. periphery regions by *t*-tests in three conscious states, including their rest and task runs, results in six *t*-tests. We applied the Bonferroni correction using a multiplication of six for all core vs. periphery comparisons or *t*-tests, respectively for the power-law exponent and mean frequency (see Supplementary Figs. 4, 5).
2. We also applied the Bonferroni correction using multiplication of three for the “Intra-region rest-task difference” of the core and periphery region, respectively, as well as for the “Inter-region rest-task difference” (see below for a detailed explanation of these computations). The Bonferroni correction with a multiplication of three results due to the three compared conscious states (three *t*-tests) in each computation.
3. The rest vs. task single region PLE analysis included seven *t*-tests for each conscious state (see below for a detailed explanation of the single region PLE analysis). We accordingly multiplied the p-values by a factor of seven.
4. Finally, we applied the Bonferroni correction with a multiplication factor of six for the computed task time windows (see Supplementary Figs. 2, 3) based on the six comparisons (two task time windows times the three conscious states).

### Core-periphery topography and seven networks

We assessed the brain’s topography via a template that divided the cerebral cortex into higher-order transmodal core and sensorimotor unimodal periphery regions. Two neuroimaging studies^23,24^ established this division based on the first principal gradient presented by refs. ^25,96,97^. The latter studies demonstrated a moderate relationship between the similarity of the gradient’s properties between two or more regions and the region’s position along the cortical surface. Paradigmatic examples of these cortical features along a gradient are functional and structural connectivity, cytoarchitecture, myeloarchitecture, gene expression, and the length of the brain’s intrinsic neuronal timescales^25,97,98^. Based on this principal gradient, the following seven networks were divided into either the core or periphery region^23,24^: default-mode (DMN), frontoparietal (FPN), dorsal attention (DAN), ventral attention (VAN), somatomotor (SMN), visual, and limbic networks^18^. The limbic, FPN, and DMN networks constitute the core region, while the visual network, SMN, DAN, and VAN constitute the periphery region^18,23,24^. It is noteworthy that the dorsal attention network (DAN) belongs to the periphery instead of the core region. While the regions’ attribution to either core or periphery is dependent on the first principal gradient in ref. ^25^, the same article shows the DAN region’s extension across unimodal sensory, such as the visual and somatomotor cortices, and across transmodal association regions, such as the frontoparietal and salience networks in Fig. 3b in ref. ^25^. We followed a previous fMRI analysis^16^ that attributed the DAN to the periphery region of the same core-periphery topography, consequently providing comparability between the findings in ref. ^18^ and our analysis.

### Power-law exponent (PLE) analysis

Increasing frequencies go along with decreasing power following a power-law function of $P=\frac{1}{{f}^{\beta }}$ where *f* is frequency, *P* is power, and the *β* is the power-law exponent (PLE)^1,2,18^. Applying the logarithm on data in the frequency domain, i.e., log(*f*) and log(*P*), displays the power-law distribution. The slope determined by a linear regression using least-square estimation between log(*f*) and log(*P*) corresponds to the PLE. The computation of the PLE was performed as follows^18^. First, AFNI’s 3dPeriodogram transformed the time-series (time-domain) into the frequency domain on a voxel-based level. We cut the resulting power spectra to the frequency band of 0.01–0.1 Hz. We chose the lower frequency limit to avoid signal contributions from scanner drift^99^, whereas the higher limit was constrained due to possible impacts from respiratory and cardiac noise. Finally, using the region-based (average) log-log transformed power spectra, we applied a linear least-square regression to estimate the PLE for each subject^18^.

### PLE control analysis I: IRASA method

To discard the possibility of the oscillatory component of the power spectrum driving our results, we applied the irregular-resampling auto-spectral analysis (IRASA) method to separate the fractal from the oscillatory component^18,20,37,100^. Briefly, the IRASA method resamples the signal with a factor *h* ranging from 1.1 to 1.9 with steps of 0.05; and $\frac{1}{h}$. Geometric means for up and downsampled PSDs were computed, then the power’s median of the geometric means across different *h* values was defined as the scale-free component. The intuition behind the method is that the scale-free component is resilient against resampling, whereas the oscillatory component is not^37^. We computed PLE levels via the slope of the linear regression fit to log-power and log frequency, but in the frequency band of 0.01–0.1 Hz. We then compared the IRASA method’s PLE levels with the conventional (empirical) PLE results using Wilcoxon tests^18^. Wilcoxon was chosen instead of *t*-tests due to the non-normality of the IRASA results tested with Kolmogorov–Smirnov tests.

### PLE control analysis II: comparison with surrogate data

To test the goodness of fit for scale-invariance, we adapted goodness of fit test for power-law distributions^39^ previously applied in fMRI studies^14,18,40,41^. For each region in rest and task states, we simulated 1000 time-series of fractional Gaussian noise (fGN) with the same length, standard deviation, and Hurst exponent as averaged time-series of real data^101^. fGN is a model of stationary scale-free dynamics^102,103^. We fitted the power-laws to each of the PSDs of synthetic time-series and real data using the maximum-likelihood estimation method^39^. Furthermore, we used Kolmogorov–Smirnov distance D to measure the goodness of fit: the larger the D, the worse the fit. *ρ*-value was defined as the fraction of synthetic time-series with Ds larger than the D of the real data. The hypothesis that the fMRI signal is scale-free was ruled out if *ρ* < 0.05. Supplementary Table 1 summarizes the surrogate data results.

### Seven networks PLE analysis

In addition to the core-periphery topography, we analyzed the PLE in all seven networks that constituted the core and periphery regions. The reason for performing the seven networks PLE computation was twofold. First, comparing the PLE in all seven networks across the three conscious states of conscious wakefulness, sedation, and anesthesia, allows controlling that the PLE systematically varied between the three conscious states on the single network level irrespective of networks’ combination into core and periphery regions. We aimed to exclude the possibility that observed PLE changes in the core and periphery regions resulted based on specific networks, e.g., by the default-mode or visual regions. Instead, we aimed to ensure that the PLE generally decreased across all or most networks in sedation and anesthesia compared to conscious wakefulness. Second, besides comparing the PLE in the seven networks across the three conscious states, the seven network PLE analysis allowed us to control that the observed PLE increases in task states in the core and periphery regions, especially under conscious wakefulness, also hold across the seven single networks.

Beyond computing the PLE in all seven networks, we aimed to control whether a hierarchy of the seven network PLE levels manifests itself under conscious wakefulness and if that hierarchy potentially diminishes in sedation and especially anesthesia. To measure this hierarchy, we computed a linear least-square regression slope across the seven networks. More precisely, we applied a one-way repeated measures ANOVA across the three conscious states in rest and task states, respectively. Beyond the statistics, we plotted the rest and task hierarchies of the seven networks for the three conscious states.

### PLE additional control analyses

Besides the IRASA (PLE control analysis I) and the comparison with surrogate data (PLE control analysis II), we investigated two additional control analyses presented in the supplement. The two control analyses include:

1. Task window analysis: We matched two time windows (volumes 90–325 and 329–564) of the task time-series to the resting-state length of 236 volumes. We subsequently computed the PLE for both windows to control that higher task-related PLE levels were not a result of the task run’s longer length (compared to the resting-state run). Supplementary Figs. 2, 3, including Supplementary Table 2, display the results.
2. Mean frequency (MF) analysis: We analyzed the power spectra’s mean frequency for all three conscious states in resting-state and task. Reporting the MF can seem contradictory, given that a fractal or scale-free process comprises no typical scale. However, systematic MF changes across the three conscious levels in rest vs. task states can back up the PLE observations. The reason for computing the MF is that it measures the balance of power between slower and faster frequencies, i.e., longer and shorter wavelengths or timescales. Hence, the PLE increase from resting-state to task under conscious wakefulness, where the brain shifts power away from faster towards slower frequencies, should be mirrored in an MF decrease. Supplementary Figs. 4,5, including Supplementary Table 3, display the results.

### Calculations of PLE rest-task differences

We calculated two additional comparisons to statistically compare rest vs. task states and core vs. periphery regions in each conscious state. Following our other computations, these calculations used the subjects’ region-based mean values. We consequently explain the exact calculations.

1. Intra-region rest-task difference: The first calculation assessed the statistical significance of the rest-to-task PLE change individually within the core and periphery regions, hence labeled intra-region rest-task difference. Paired *t*-tests between rest vs. task PLE levels tested for intra-region statistical significance. The first calculation allowed us to check if the intra-region rest-to-task PLE change remains significant in sedation and anesthesia.
2. Inter-region rest-task difference: The second calculation assessed the absolute rest-to-task PLE change in the core and periphery regions, respectively. First, we subtracted the task from the rest PLE levels, individually for the core and periphery regions. Second and contrary to the first step no longer within only one region, we statistically compared the absolute PLE change between core and periphery regions using paired *t*-tests, hence labeling this calculation inter-region rest-task difference. The second calculation controlled if the periphery region exhibits significantly higher task-related PLE changes than the core region.

### Computational Stuart–Landau model

To investigate the effects of different inputs on a system of coupled oscillators, we used the Stuart–Landau model (Eqs. 1 and 2)^32–34^:

1 $$\frac{{dx}}{{dt}}=\lambda x-\omega y-({x}^{2}+{y}^{2})x+I(t)$$

2 $$\frac{{dy}}{{dt}}=\lambda x-\omega y-({x}^{2}+{y}^{2})y$$

where *x* and *y* are the two oscillators near Hopf–Andronov bifurcation^31^ and *λ* is the coupling parameter. For $\lambda > 0$, the two oscillators oscillate synchronously with frequency $\omega$ whereas for $\lambda < 0$, the system decays to a stable equilibrium. For all simulations, we set *λ* to 0.1 and $\omega$ to 0.05 and changed the *I* which is the input to the system. We provided white, pink, and brown noises (see below) scaled between input strengths 0.1, 1, and 10 to see their effect on synchronization. The differential equations were integrated with the Euler method with 0.1 milisecond steps for 100 s. The first 50 s were discarded to get the steady state. We used MATLAB’s dsp.ColoredNoise function from DSP System Toolbox to generate different inputs based on ref. ^101^. Gaussian white noise is colored by multiplication with an autoregressive model of order 63 in the frequency domain to filter its power spectrum according to the power law $P={f}^{\beta }$. Three different input scenarios were simulated: white noise ($\beta =0$) corresponding to no temporal correlations, pink noise ($\beta =-1$) corresponding to intermediate temporal correlations, and brown noise ($\beta =-2$) corresponding to high temporal correlations. Simulations were repeated 50 times.

Quantification of Perturbation: In the absence of any external stimulus, the two oscillators form a circle in the phase space. To compare the disturbance caused by noise, we took the absolute value of the difference in the surface area in the phase space after external stimulation. The surface area was calculated with the MATLAB function polyarea. In Fig. 6b top, we provided white, pink, and brown noises to the system with linearly increasing noise strengths from 0.1 to 100 in 20 simulations. On the bottom, we provided three different inputs strengths of 10, 30, and 50 with different noise colors from 0 (white noise) to 2 (brown noise).

### Reporting summary

Further information on research design is available in the Nature Portfolio Reporting Summary linked to this article.

## Supplementary information

Supplementary information

Description of Additional Supplementary Files

Supplementary Data 1

Reporting Summary

## Data Availability

The functional MRI dataset assessed in this analysis is available from the corresponding author upon reasonable request.

## Code availability

The code to replicate the Stuart–Landau model analysis is publicly available at https://github.com/duodenum96/anesthesia_SL.
