# Supplementary material for: Scale-free dynamics in the core-periphery topography and task alignment decline from conscious to unconscious states
Source: Commun Biol. 2023 May 9;6:499. doi: 10.1038/s42003-023-04879-y (PMC10170069; doi:10.1038/s42003-023-04879-y)
Supplement: Supplementary file 1 — Supplementary information [file 42003_2023_4879_MOESM1_ESM.pdf]

# Supplement for "Scale-free dynamics in the core-periphery topography and task alignment decline from conscious to unconscious states"

## Overview of the control analyses

The functional magnetic resonance imaging (fMRI) analysis investigated scale-free dynamics across three conscious states of conscious wakefulness, sedation, and anesthesia-induced unconsciousness in the core-periphery topography of the human cerebral cortex. We operationalized scale-free dynamics by assessing the log-log power spectra's inverse power-law distributions' slope, namely the power-law exponent (PLE). Our replication and control analyses assessed the PLE in the same dataset[1] that we used for the primary findings of our fMRI analysis. After presenting the control analyses, this supplementary file summarizes results from the main manuscript in Tables 4, 5, and 6. Besides the second PLE control analysis using surrogate data (see section PLE Control analysis II: Comparison with surrogate data), our control analyses include two control analyses to back up the observed PLE levels in the three conscious levels, including the PLE change from rest and task states.

(1) We computed two time windows (volumes 90-325 and 329-564) of the task time-series matched to the length of the resting-state run (236 volumes). Assessing the PLE in the two time windows allowed us to check if significant PLE differences between core and periphery regions, as observed in the resting-state, also vanish in the shorter time windows (as observed in the full-length task runs). Additionally, we controlled that task-related PLE increases were not an artifact induced by the task run's longer recording times. Supplementary Figures 2 and 3, including Supplementary Table 2, display the results.

(2) Mean frequency (MF) analysis: We analyzed the power spectra's mean frequency for all three conscious levels in rest and task states. Reporting the MF can seem contradictory, given that a fractal or scale-free process comprises no typical scale. However, systematic MF changes across the three conscious levels in rest vs. task states can back up the PLE observations. The reason for computing the MF is that it measures the balance of power between slower and faster frequencies, i.e., longer and shorter wavelengths or timescales. Hence, the PLE increase from resting-state to task under conscious wakefulness, where the brain shifts power away from faster towards slower frequencies, should be mirrored in an MF decrease. Supplementary Figures 4 and 5, including Supplementary Table 3, display the results.

## PLE control and replication analyses

PLE control analysis I: distinction between fractal and oscillatory components (IRASA): The IRASA method[2] was applied to separate oscillatory and fractal components of the power spectrum, previously successfully measured in electroencephalography (EEG) and magnetoencephalography (MEG) recordings[2,3,4]. Supplementary Figure 1 displays the comparison between the conventionally computed PLE presented above, including both fractal and oscillatory components in the power spectrum, and the IRASA method obtained fractal-based PLE values (exclusion of oscillatory components) in rest and task states. The comparison between both analysis methods yielded no significant differences. The results indicate that our PLE results were not driven by oscillatory components, but reflected a genuine change in the power spectras' fractal component, that is, in scale-free dynamics.

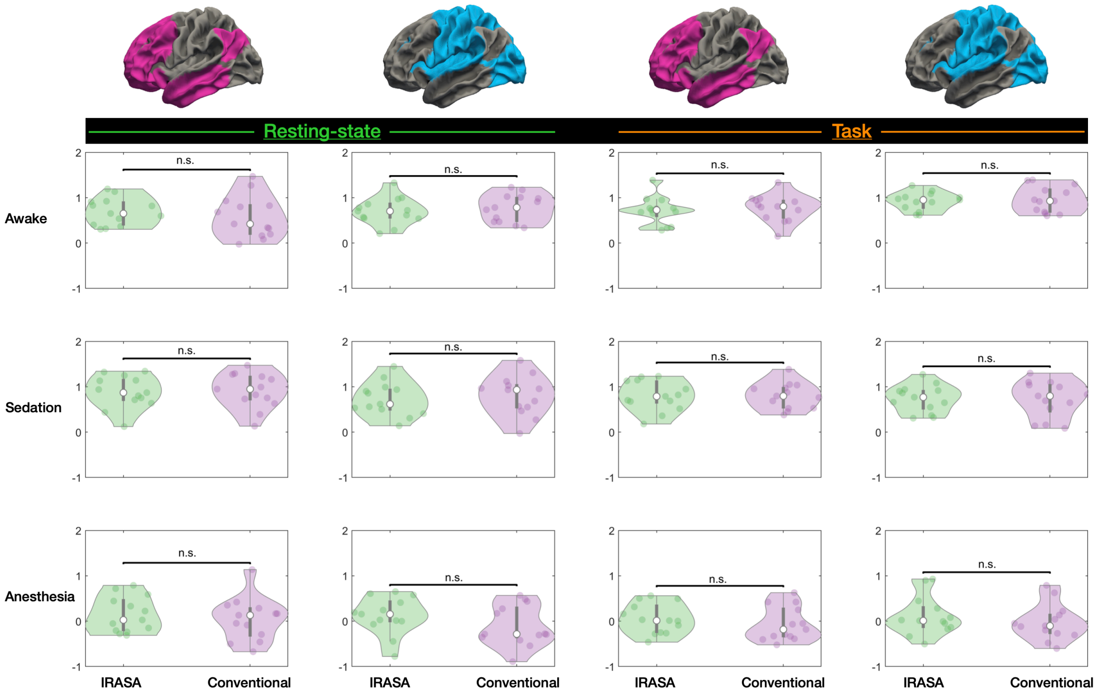


**Supplementary Figure 1.** IRASA method and results. PLE values computed via the IRASA method (separation between fractal and oscillatory components) and the conventional method (no separation between fractal and oscillatory components) across all conscious levels in rest and task states. No significant differences were obtained in any of the comparisons.

## PLE Control analysis II: Comparison with surrogate data

We tested wherever the measured power spectra were scale-free by comparing the goodness of fit of the power-law to the PSDs of real data and simulated fractional Gaussian noise (fGn)[5,6,7,8,9]. The core and periphery regions had $p$-values, that is, the fraction of synthetic time-series that had a worse fit than real data, exceeded 0.05, except the awake task in the periphery region (0.021) and anesthesia task in the core region (0.045). The results provided further evidence for genuine scale-free dynamics in the awake state and are displayed in Supplementary Table 1.

**Supplementary Table 1**
PLE Control Analysis with surrogate data

| Conscious state | Run | Region | *p*-value |
|---|---|---|---|
| Awake (Propofol 0 µg/ml; Ramsay 1.0 ± 0) | Rest | Core | 0.288 |
| | Rest | Periphery | 0.122 |
| | Task | Core | 0.942 |
| | Task | Periphery | 0.021 * |
| Sedation (Propofol 1.3 µg/ml; Ramsay 3.8 ± 1.7) | Rest | Core | 0.197 |
| | Rest | Periphery | 0.227 |
| | Task | Core | 0.289 |
| | Task | Periphery | 0.797 |
| Anesthesia (Propofol 4.0 µg/ml; Ramsay 6 ± 0) | Rest | Core | 0.285 |
| | Rest | Periphery | 0.161 |
| | Task | Core | 0.045 * |
| | Task | Periphery | 0.799 |

Significance asterisks, $p < 0.05$ *, $p < 0.01$ ** , $p < 0.001$ ***, $n = 13$ subjects.

## 1. Two task time windows to control different lengths between rest and task runs

We computed two time windows of the task's time-series matched to the resting-state length of 236 volumes. Task window one contained volumes 90-325, and task window two volumes 329-574. The creation of both time windows included the same number of self- and non-self-related trials (time window one = 13 self and 13 non-self; time window two = 14 self and 14 non-self). Due to the trials' given onset times and the constraint of 236 sampling points to match the resting-state length, choosing the same trial number in both time windows remained impossible. We subsequently computed the PLE in both time windows. Compared to the resting-state, both time windows showed the same PLE increase and convergence between core and periphery regions, mirroring the results we previously observed in the full-length task run of our primary analysis. Supplementary Figures 2 and 3, including Supplementary Table 2, summarize the task windows' results.

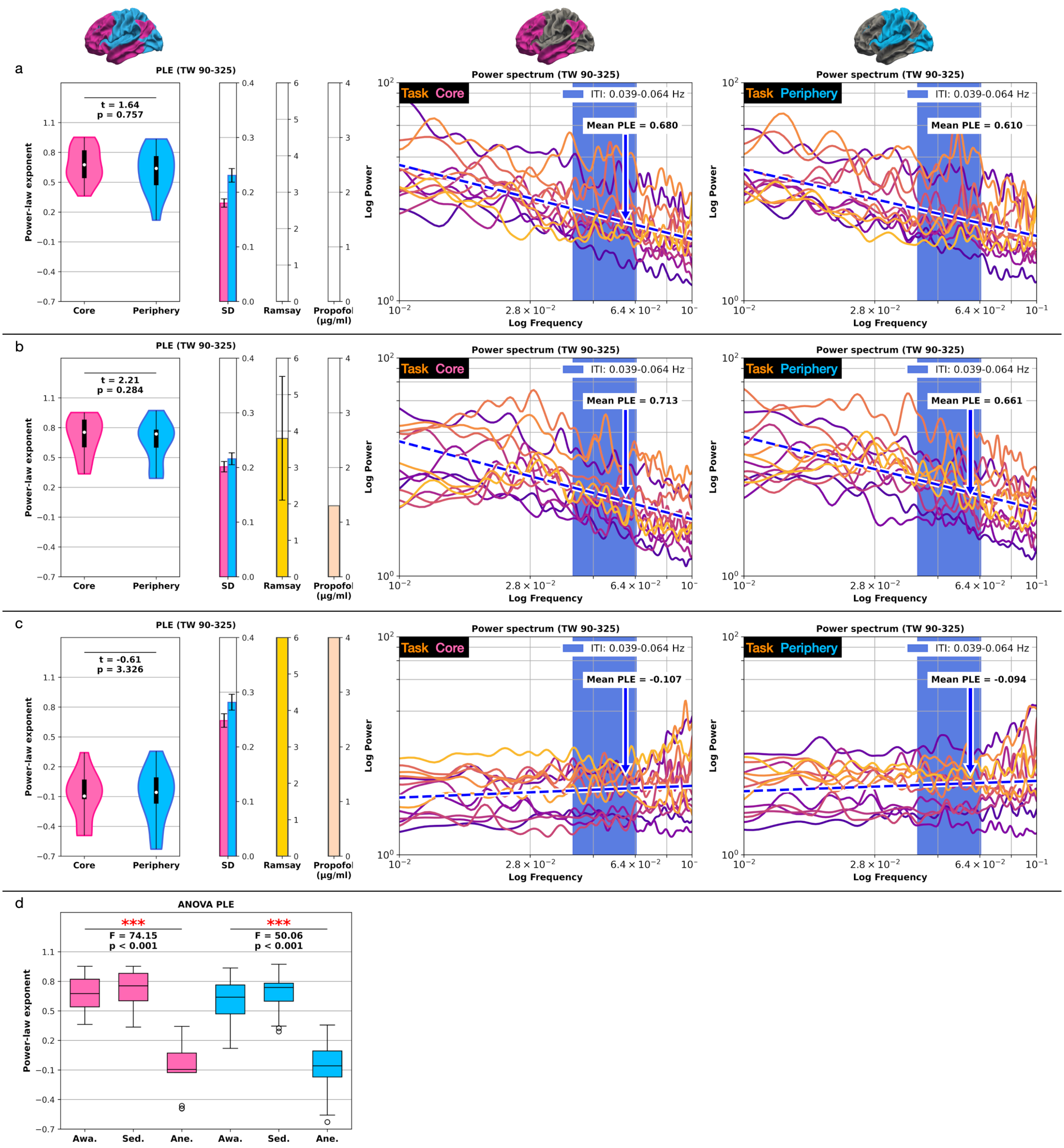


**Supplementary Figure 2.** First time window (volumes 90-325) inverse power-law distributions and PLE where each line represents one subject. The blue dashed line represents the mean linear least-square regression across all subjects.

The blue shaded area represents the task's frequency range (0.039-0.064 Hz). **a)** The core-periphery comparison lacked significant PLE differences under conscious wakefulness. **b)** The PLE further decreased and converged between the core and periphery regions in sedation. **c)** The power spectra flattened to white noise under anesthesia/ unconsciousness (0.01-0.1 Hz or $10^{-2}$ to $10^{-1}$ on the logarithmic scale). **d)** One-way repeated measures ANOVA between the three states of consciousness. Error bars for the SD represent the standard error of the mean (SEM) based 599 bootstrap samples and SD for the Ramsay score. Boxplot center line represents the median, boxes the interquartile range (IQR), and whiskers 1.5x IQR; *n* = 13 subjects. (PLE, power-law exponent; SD, Standard deviation; Awa, awake; Sed, sedation, Ane, anesthesia.)

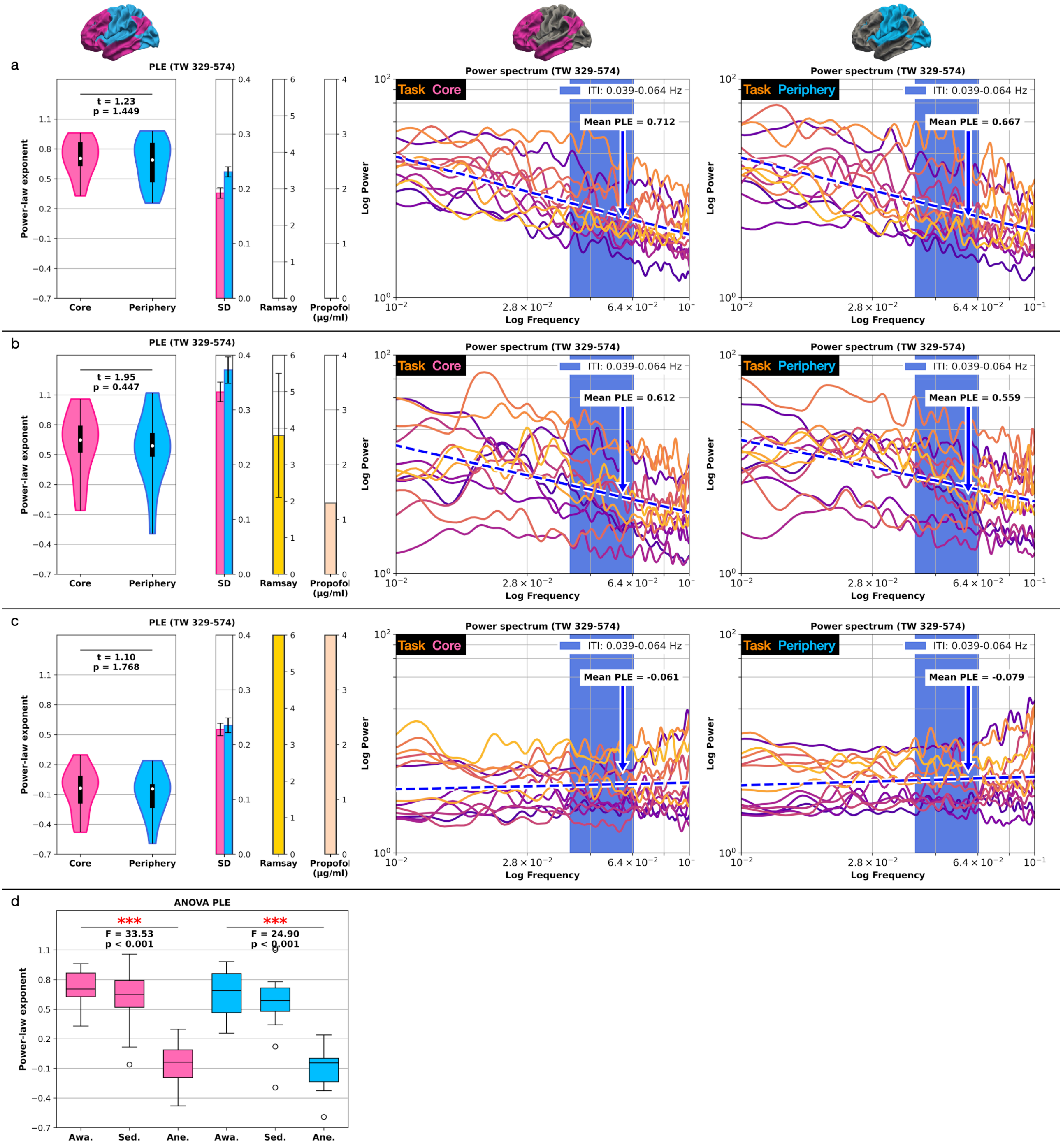


**Supplementary Figure 3.** First time window (volumes 329-574) inverse power-law distributions and PLE where each line represents one subject. The blue dashed line represents the mean linear least-square regression across all subjects. The blue shaded area represents the task's frequency range (0.039-0.064 Hz). **a)** The core-periphery comparison lacked significant PLE differences under conscious wakefulness. **b)** The PLE further decreased and converged between the core and periphery regions in sedation. **c)** The power spectra flattened to white noise under anesthesia/ unconsciousness (0.01-0.1 Hz or $10^{-2}$ to $10^{-1}$ on the logarithmic scale). **d)** One-way repeated measures ANOVA between the three states of consciousness. Error bars for the CV and SD represent the standard error of the mean (SEM) based

599 bootstrap samples and SD for the Ramsay score. Boxplot center line represents the median, boxes the interquartile range (IQR), and whiskers 1.5x IQR; *n* = 13 subjects. (PLE, power-law exponent; SD, Standard deviation; Awa, awake; Sed, sedation, Ane, anesthesia.)

**Supplementary Table 2**
Power-law exponent (PLE) core-periphery comparison in two task time windows

| Conscious state | Time window | Core | Periphery | *t*-value | *p*-value |
|---|---|---|---|---|---|
| Awake (Propofol 0 μg/ml; Ramsay 1.0 ± 0) | 90-325 | 0.68 (0.18) | 0.610 (0.231) | 1.64 | 0.757 |
| | 329-574 | 0.712 (0.192) | 0.667 (0.231) | 1.23 | 1.449 |
| Sedation (Propofol 1.3 μg/ml; Ramsay 3.8 ± 1.7) | 90-325 | 0.713 (0.201) | 0.661 (0.216) | 2.21 | 0.284 |
| | 329-574 | 0.612 (0.333) | 0.559 (0.373) | 1.95 | 0.447 |
| Anesthesia (Propofol 4.0 μg/ml; Ramsay 6 ± 0) | 90-325 | -0.107 (0.248) | -0.094 (0.282) | -0.61 | 3.326 |
| | 329-574 | -0.061 (0.228) | -0.079 (0.235) | 1.10 | 1.768 |

Data represents ROI-based mean values including the standard deviation (SD) in brackets. Time window values represent the respective volumes. Statistics, Student's paired *t*-Test where *p*-values are multiplied by six (Bonferroni correction); PLE, power-law exponent; significance asterisks, $p < 0.05$ *, $p < 0.01$ ** , $p < 0.001$ ***; *n* = 13 subjects.

## Mean frequency (MF) analysis

We computed the power spectra's mean frequency (MF) for all three conscious states (conscious wakefulness, sedation, and anesthesia). Computation of the MF used AFNI's 3dPeriodogram to compute the power spectrum on a voxel-based level. The MF computation used the same frequency band (0.01-0.1 Hz) previously applied for the PLE. In a second step, AFNI's 3dTstat computed both (1) the sum of power multiplied by frequency and (2) the sum of power. We divided the power times frequency by the power. Consequently, only the mean frequency per voxel remains.

Reporting the MF can seem contradictory, given that a fractal or scale-free process comprises no typical scale. However, systematic MF changes across the three conscious levels in rest vs. task states can back up the PLE observations. The reason for computing the MF is that it measures the balance of power between slower and faster frequencies, i.e., longer and shorter wavelengths or timescales. Hence, the PLE increase from resting-state to task under conscious wakefulness, where the brain shifts power away from faster towards slower frequencies, should be mirrored in an MF decrease. Conversely, flattening power spectra under propofol-induced anesthesia, i.e., lower PLE levels, should correspond to increasing MF levels. When comparing rest vs. task states, the MF should decrease in response to the task's infra-slow periodicity. The MF decrease in task states is predicted to appear especially under conscious wakefulness, less so in sedation, and absent in anesthesia. We subsequently present the MF results.

Overall, the MF results followed our predictions and the PLE results. Supplementary Figures 4 and 5 display the rest and task state MF results, respectively. Supplementary Table 3 summarizes the results.

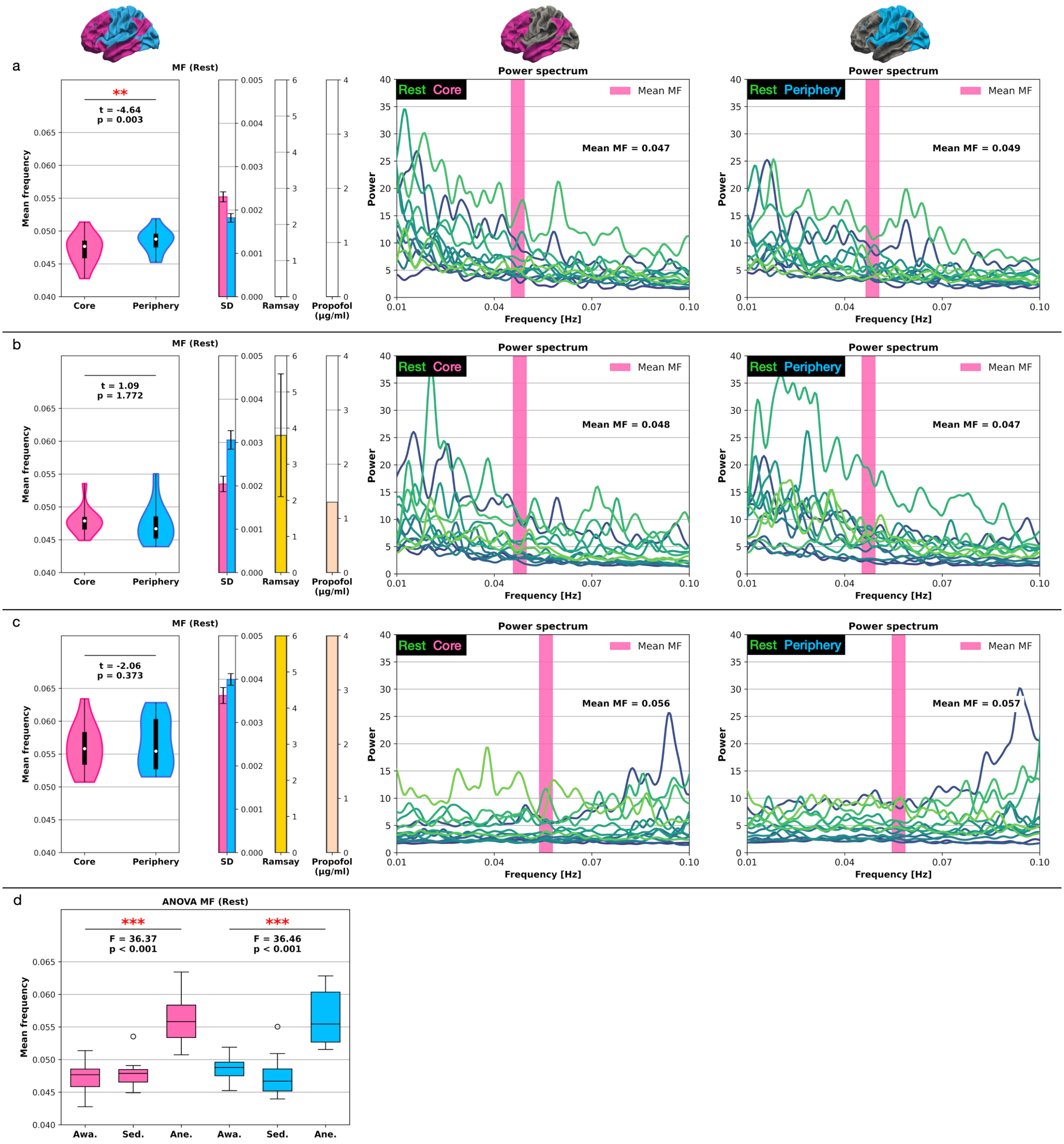


**Supplementary Figure 4.** Power spectra and MF, where each line represents one subject. **a)** The core-periphery comparison yielded significant MF differences under conscious wakefulness. **b)** The MF converged between the core and periphery regions in sedation. **c)** MF converged and increased further under anesthesia-induced unconsciousness. **d)** One-way repeated measures ANOVA between the three states of consciousness. Vertical bars in the power spectra represent the mean frequency. MF, mean frequency; SD, standard deviation. Error bars for the SD represent the standard error of the mean (SEM) based 599 bootstrap samples and SD for the Ramsay score; *n* = 13 subjects.

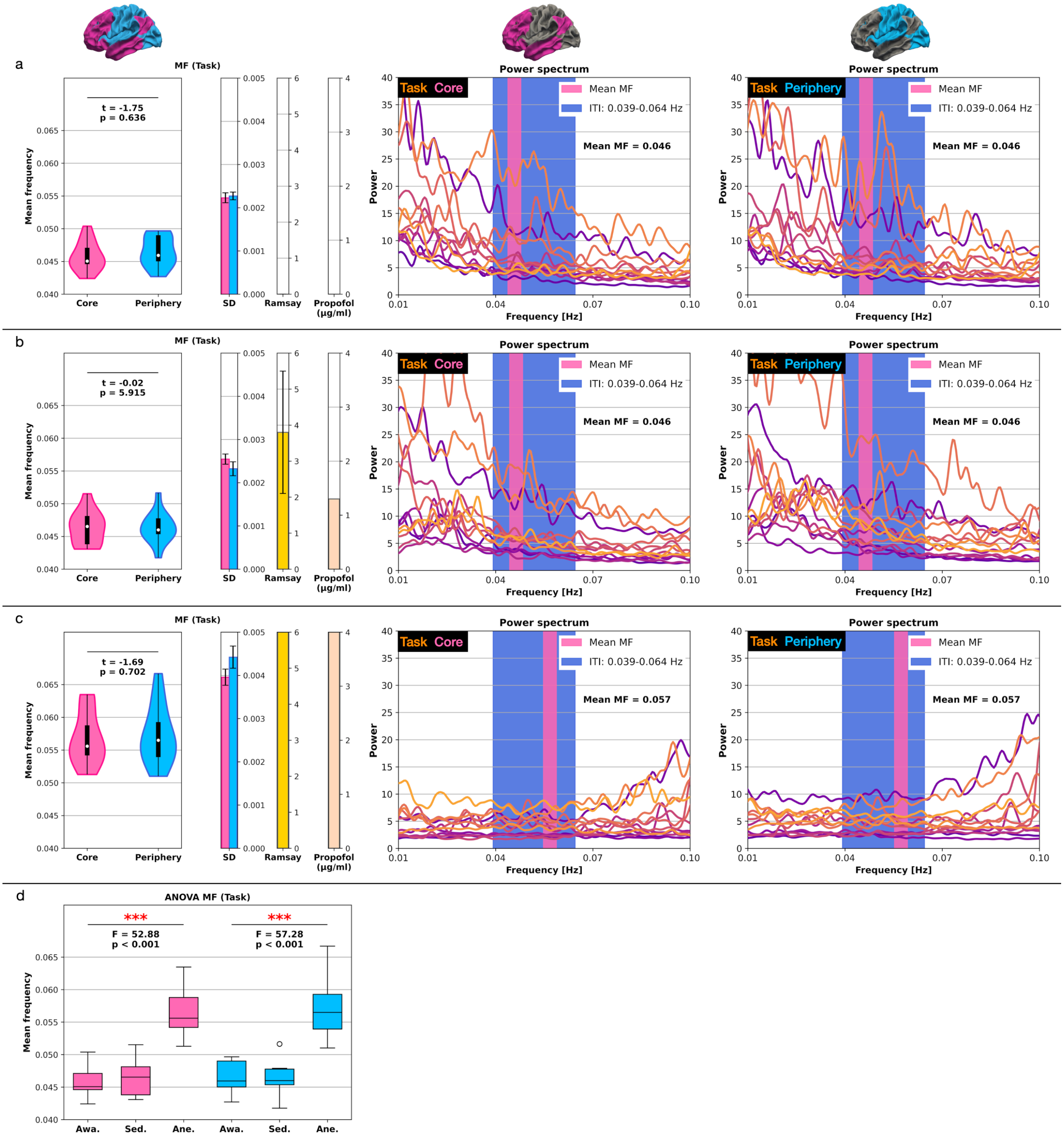


**Supplementary Figure 5.** Power spectra and MF, where each line represents one subject. **a)** The core-periphery comparison yielded no significant MF differences under conscious wakefulness. **b)** The MF in sedation. **c)** MF increased under anesthesia-induced unconsciousness. **d)** One-way repeated measures ANOVA between the three states of consciousness. Vertical bars in the power spectra represent the mean frequency and inter-trial interval (15.5-25.5 s; 0.039-0.064 Hz). MF, mean frequency; SD, standard deviation. Error bars for the SD represent the standard error of the mean (SEM) based 599 bootstrap samples and SD for the Ramsay score; *n* = 13 subjects.

**Supplementary Table 3**
Mean frequency (MF) core-periphery comparison

| Conscious state | Run | Core | Periphery | *t*-value | *p*-value |
|---|---|---|---|---|---|
| Awake (Propofol 0 µg/ml; Ramsay 1.0 ± 0) | Rest | 0.0472 (0.0023) | 0.0485 (0.0018) | -4.64 | 0.003 |
| | Task | 0.0458 (0.0022) | 0.0465 (0.0023) | -1.75 | 0.636 |
| Sedation (Propofol 1.3 µg/ml; Ramsay 3.8 ± 1.7) | Rest | 0.0479 (0.002) | 0.0473 (0.0031) | 1.09 | 1.772 |
| | Task | 0.04631 (0.0025) | 0.0463 (0.0023) | -0.02 | 5.915 |
| Anesthesia (Propofol 4.0 µg/ml; Ramsay 6 ± 0) | Rest | 0.05591 (0.0036) | 0.05656 (0.004) | -2.06 | 0.373 |
| | Task | 0.0568 (0.004) | 0.0573 (0.0044) | -1.69 | 0.702 |

Data represents ROI-based mean values across subjects including the standard deviation (SD) in brackets. Statistics, Student's paired *t*-Test where *p*-values are multiplied by six (Bonferroni correction); significance asterisks, $p < 0.05$ *, $p < 0.01$ ** , $p < 0.001$ ***; $n = 13$ subjects.

The three Supplementary Tables 4, 5, and 6 shown subsequently summarize results from the main manuscript.

**Supplementary Table 4**
Resting-state and Task power-law exponent (PLE) ANOVA

| Run | Region | F-value | *p*-value |
|---|---|---|---|
| Rest | Core | 40.38 | p < 0.001 *** |
| | Periphery | 46.1 | p < 0.001 *** |
| Task | Core | 62.79 | p < 0.001 *** |
| | Periphery | 47.64 | p < 0.001 *** |

Statistics, one-way repeated measures ANOVA; significance asterisks, $p < 0.05$ *, $p < 0.01$ ** , $p < 0.001$ ***; $n = 13$ subjects.

**Supplementary Table 5**
Seven networks power-law exponent (PLE) in rest and task states

| Conscious state | Region | Rest | Task | *t*-value | *p*-value |
|---|---|---|---|---|---|
| Awake (Propofol 0 μg/ml; Ramsay 1.0 ± 0) | Visual | 0.550 | 0.678 | 2.57 | 0.170 |
| | SMN | 0.355 | 0.470 | 2.88 | 0.097 |
| | DAN | 0.588 | 0.719 | 3.90 | 0.015 * |
| | VAN | 0.462 | 0.508 | 0.96 | 2.478 |
| | Limbic | 0.515 | 0.623 | 1.68 | 0.829 |
| | FPN | 0.646 | 0.734 | 4.49 | 0.005 ** |
| | DMN | 0.639 | 0.691 | 2.40 | 0.234 |
| Sedation (Propofol 1.3 μg/ml; Ramsay 3.8 ± 1.7) | Visual | 0.631 | 0.661 | 0.32 | 5.267 |
| | SMN | 0.529 | 0.575 | 0.63 | 3.793 |
| | DAN | 0.672 | 0.694 | 0.28 | 5.504 |
| | VAN | 0.459 | 0.530 | 1.21 | 1.744 |
| | Limbic | 0.359 | 0.544 | 2.59 | 0.166 |
| | FPN | 0.620 | 0.706 | 1.27 | 1.594 |
| | DMN | 0.628 | 0.707 | 1.08 | 2.098 |
| Anesthesia (Propofol 4.0 μg/ml; Ramsay 6 ± 0) | Visual | -0.055 | -0.056 | -0.02 | 6.906 |
| | SMN | -0.102 | -0.129 | -0.67 | 3.609 |
| | DAN | -0.044 | -0.047 | -0.07 | 6.612 |
| | VAN | -0.123 | -0.122 | 0.03 | 6.842 |
| | Limbic | -0.035 | -0.067 | -0.78 | 3.165 |
| | FPN | -0.034 | -0.054 | -0.47 | 4.541 |
| | DMN | -0.049 | -0.094 | -0.94 | 2.546 |

Data represents network-based mean values across subjects. Statistics = Student's paired *t*-Test where p-values are multiplied by seven (Bonferroni correction). Significance asterisks, $p < 0.05$ *, $p < 0.01$ ** , $p < 0.001$ ***; $n = 13$ subjects. (DMN, default-mode network; FPN, fronto-parietal network; DAN, dorsal attention network; VAN, ventral attention network; SMN, somatomotor network.)

**Supplementary Table 6**
Resting-state and Task mean slopes and ANOVA for the seven network PLE hierarchy

| | Rest | Task |
|---|---|---|
| Awake | 0.044 | 0.039 |
| Sedation | 0.037 | 0.031 |
| Anesthesia | 0.009 | 0.007 |
| F-value | 4.98 | 4.15 |
| *p*-value | 0.015 * | 0.028 * |

Statistics, one-way repeated measures ANOVA; significance asterisks, $p < 0.05$ *, $p < 0.01$ ** , $p < 0.001$ ***; $n = 13$ subjects.

## Supplementary References

1. Huang, Z. et al. Disrupted neural variability during propofol-induced sedation and unconsciousness. *Hum. Brain Mapp.* **39(11)**, 4533–4544 (2018).
2. Wen, H., Liu, Z. Separating Fractal and Oscillatory Components in the Power Spectrum of Neurophysiological Signal. *Brain Topogr.* **29(1)**, 13-26 (2016).
3. Wainio-Theberge, S., Wolff, A., Northoff, G. Dynamic relationships between spontaneous and evoked electrophysiological activity. *Commun. Biol.* **4(1)**:741 (2021).
4. Wainio-Theberge, S., Wolff, A., Gomez-Pilar, J., Zhang, J., Northoff, G. Variability and task-responsiveness of electrophysiological dynamics: Scale-free stability and oscillatory flexibility. *NeuroImage* **256,** 119245 (2022).
5. Clauset, A., Shalizi, C. R., Newman, M. E. J. Power-law distributions in empirical data. *SIAM Rev.* **51(4)**, 661-703; https://doi.org/10.1137/07071011 (2009).
6. He, B. J. Scale-free properties of the functional magnetic resonance imaging signal during rest and task. *J Neurosci.* **31(39)**, 13786–13795 (2011).
7. Tagliazucchi, E., von Wegner, F., Morzelewski, A., Brodbeck, V., Jahne, K., Laufs, H. Breakdown of long-range temporal dependence in default mode and attention networks during deep sleep. Proc. Natl. Acad. Sci. **110(38)**, 15419-15424 (2013).
8. Scalabrini, A. et al. How spontaneous brain activity and narcissistic features shape social interaction. Sci. Rep. **7(1)**, 9986; https://doi.org/10.1038/s41598-017-10389-9 (2017).
9. Çatal, Y., Gomez-Pilar, J., Northoff, G. Intrinsic Dynamics and Topography of Sensory Input Systems. Cereb. Cortex **32(20)**, 4592-4604 (2022).
