# Supplementary material for: Scale-free dynamics in the core-periphery topography and task alignment decline from conscious to unconscious states
Source: Commun Biol. 2023 May 9;6:499. doi: 10.1038/s42003-023-04879-y (PMC10170069; doi:10.1038/s42003-023-04879-y)
Supplement: Supplementary file 2 — Description of Additional Supplementary Files [file 42003_2023_4879_MOESM2_ESM.pdf]

Description of Additional Supplementary Files

File name: Supplementary Data 1

Description: The source data results underlying the figures
