# Supplementary material for: Scale-free dynamics in the core-periphery topography and task alignment decline from conscious to unconscious states
Source: Commun Biol. 2023 May 9;6:499. doi: 10.1038/s42003-023-04879-y (PMC10170069; doi:10.1038/s42003-023-04879-y)
Supplement: Supplementary file 3 — Supplementary Data 1 [file 42003_2023_4879_MOESM3_ESM.pdf]

Sessions	Rest Awake			
Variables and ROIs	PLE Core	PLE Periphery	MF Core	MF Periphery
Subject1	0.5342771	0.5666602	0.0485344	0.0475075
Subject3	0.7799943	0.5957555	0.0458402	0.0473261
Subject5	0.4531863	0.3987293	0.0494248	0.0498344
Subject6	0.7012888	0.5090784	0.0456482	0.0484867
Subject7	0.7020954	0.5159787	0.0467135	0.0487694
Subject8	0.8100513	0.7140739	0.0443322	0.0455472
Subject9	0.5616336	0.5194375	0.0476692	0.0494339
Subject10	0.7557886	0.644011	0.0459215	0.0480155
Subject11	0.9644683	0.8340158	0.0427675	0.0452342
Subject12	0.5099727	0.4200479	0.0487306	0.0498156
Subject13	0.5406156	0.3955894	0.0484716	0.0493101
Subject14	0.5366849	0.3672039	0.0484062	0.0495898
Subject15	0.3427445	0.2487708	0.0513613	0.0518785
**Mean value**	0.630215492307692	0.517642484615385	0.0472170153846154	0.0485191461538462
**Standard deviation (SD)**	0.171174201936567	0.15724605059468	0.00230357510471800	0.00181870116640775
**Coefficient of Variation (CV)**	0.271612177145582	0.303773463863801	0.0487869698233541	0.0374841956336325
**Sessions**	**Rest Sedation**			
**Variables and ROIs**	**PLE Core**	**PLE Periphery**	**MF Core**	**MF Periphery**
Subject1	0.6230807	0.6437804	0.0478613	0.0466743
Subject3	0.6729651	0.4976846	0.0464251	0.0485782
Subject5	0.5635185	0.6196858	0.0485355	0.0485536
Subject6	0.8216872	0.8343742	0.0449024	0.0439576
Subject7	0.762469	0.8162248	0.046373	0.0446316
Subject8	0.6102731	0.4244814	0.0484687	0.0509104
Subject9	0.7048112	0.6070358	0.0473488	0.0478136
Subject10	0.6394319	0.7261902	0.0471565	0.0445332
Subject11	0.4347635	0.5826306	0.0480562	0.0476765
Subject12	0.7101329	0.6629527	0.0465442	0.0452535
Subject13	0.0977709	0.0119784	0.0535427	0.0550343
Subject14	0.5444197	0.7098122	0.0479474	0.0451743
Subject15	0.5269366	0.6602165	0.0490739	0.046425
**Mean value**	0.593250792307692	0.599772892307692	0.0478642846153846	0.0473243153846154
**Standard deviation (SD)**	0.181401120020249	0.209554207222505	0.00204418722187490	0.00305871174893560
**Coefficient of Variation (CV)**	0.305774762330472	0.349389260351901	0.0427079865143942	0.0646329846311943
**Sessions**	**Rest Anesthesia**			
**Variables and ROIs**	**PLE Core**	**PLE Periphery**	**MF Core**	**MF Periphery**
Subject1	0.0232394	0.015828	0.0541321	0.0544746
Subject3	-0.5263357	-0.4282841	0.063411	0.0628284
Subject5	0.3561596	0.2659971	0.0507302	0.0515515
Subject6	-0.2358781	-0.332862	0.0583425	0.0592859
Subject7	-0.1819825	-0.1086768	0.0545987	0.0554394
Subject8	-0.0832032	-0.1292218	0.0565673	0.0572264
Subject9	-0.0987054	-0.2759267	0.0573647	0.0603322
Subject10	-0.0273579	0.0260691	0.055816	0.0552296
Subject11	0.1925058	0.1986068	0.0533656	0.0525039
Subject12	-0.212816	-0.2966225	0.0596908	0.0612614
Subject13	-0.3341323	-0.4026704	0.0586882	0.0608761
Subject14	0.1771375	0.1908487	0.0531768	0.0526727
Subject15	0.2749294	0.227887	0.0509119	0.0515387
**Mean value**	-0.0520338	-0.0806944307692300	0.0559073692307692	0.0565554461538462
**Standard deviation (SD)**	0.254353555484045	0.252294713895695	0.00362155541961010	0.00399337721392460
**Coefficient of Variation (CV)**	-4.88823717437598	-3.12654431651182	0.0647777827760316	0.0706099498015017

Sessions	Task Awake			
Variables and ROIs	PLE Core	PLE Periphery	MF Core	MF Periphery
Subject1	0.8309851	0.8375008	0.0447636	0.0446669
Subject3	0.8412524	0.6528896	0.0437097	0.0450081
Subject5	0.5057871	0.449079	0.0484478	0.0493238
Subject6	0.7624762	0.6412688	0.0450511	0.0469864
Subject7	0.716903	0.6870016	0.0459433	0.0459297
Subject8	0.8705176	0.8507273	0.043655	0.0438044
Subject9	0.7146909	0.5503258	0.0445883	0.0475009
Subject10	0.7991422	0.8124777	0.0449676	0.0451461
Subject11	0.9299532	0.9494711	0.0424026	0.042698
Subject12	0.5012532	0.4481239	0.0481856	0.049
Subject13	0.5981666	0.658452	0.0470894	0.045704
Subject14	0.6036554	0.1802068	0.0458483	0.0496582
Subject15	0.3844181	0.4333065	0.0503804	0.0490384
Mean value	0.696861615384615	0.626986992307692	0.0457717461538462	0.0464973
Standard deviation (SD)	0.165739053273994	0.213090391049849	0.0022298323466931	0.0022726494655651
Coefficient of Variation (CV)	0.237836393359847	0.339864133808498	0.0487163487099292	0.048877020075684
Sessions	Task Sedation			
Variables and ROIs	PLE Core	PLE Periphery	MF Core	MF Periphery
Subject1	0.9984797	1.0063934	0.0437169	0.0417552
Subject3	0.7333051	0.6436939	0.0448621	0.0460778
Subject5	0.6366093	0.603533	0.0463255	0.0477613
Subject6	0.8804533	0.755034	0.0430737	0.0446293
Subject7	0.6631733	0.6912655	0.0473504	0.0453495
Subject8	0.99466	1.044297	0.0431067	0.0453543
Subject9	0.2503756	0.2179708	0.051514	0.0516379
Subject10	0.4630871	0.4417232	0.0489759	0.0477686
Subject11	0.5390231	0.4231386	0.0481342	0.0478855
Subject12	0.8630637	0.6946024	0.0438078	0.0448732
Subject13	0.5949251	0.5488839	0.0465494	0.0475295
Subject14	0.6661674	0.55614	0.0465238	0.0459934
Subject15	0.6836053	0.7137444	0.0481168	0.0455444
Mean value	0.689763692307692	0.641570776923077	0.0463120923076923	0.0463199923076923
Standard deviation (SD)	0.211511822422741	0.224179629568259	0.0025459586346016	0.0023237891977908
Coefficient of Variation (CV)	0.306643890917339	0.349423068555907	0.054973949734047	0.0501681689054359
Sessions	Task Anesthesia			
Variables and ROIs	PLE Core	PLE Periphery	MF Core	MF Periphery
Subject1	-0.0701876	-0.125734	0.0555836	0.0564893
Subject3	-0.4414416	-0.306566	0.0633466	0.0619042
Subject5	0.2786664	0.3229832	0.051269	0.0510013
Subject6	-0.1773109	-0.1995552	0.0574956	0.0578744
Subject7	-0.0454221	0.0329027	0.0528685	0.0531675
Subject8	-0.0752592	-0.0787666	0.0562744	0.0566299
Subject9	-0.4531792	-0.6038392	0.0634692	0.0666698
Subject10	0.1501517	0.1296628	0.0547445	0.0550236
Subject11	0.0924983	0.1330029	0.0541809	0.0539202
Subject12	-0.0871711	-0.0601922	0.0587753	0.0592599
Subject13	-0.4372025	-0.4089473	0.0614869	0.0628729
Subject14	0.0873964	0.0737837	0.0533087	0.0535579
Subject15	0.0442057	-0.0042088	0.0549832	0.0558816
Mean value	-0.087250438461538	-0.0842672307692308	0.0567528	0.0572501923076923
Standard deviation (SD)	0.234958128806668	0.249057074632601	0.0039585264503768	0.0044221561425511
Coefficient of Variation (CV)	-2.69291631021707	-2.9555625877235	0.069750328624788	0.07724264258859

Session	Awake													
Runs	Rest	Task	Rest	Task	Rest	Task	Rest	Task	Rest	Task	Rest	Task	Rest	Task
Variables and ROIs	PLE Visual		PLE SMN		PLE DAN		PLE VAN		PLE Limbic		PLE FPN		PLE DMN	
Subject1	0.5334069	1.0095274	0.4334227	0.5614843	0.8838563	0.8956588	0.3359629	0.7243586	0.1820445	1.0144382	0.6783343	0.7875921	0.5333179	0.7900674
Subject3	0.6121537	0.5994518	0.4246027	0.5203745	0.708334	0.7979944	0.5316901	0.7424549	0.4679573	0.5049781	0.8879785	0.9268506	0.7610203	0.8473763
Subject5	0.4950049	0.5566067	0.3597531	0.3783344	0.3452932	0.4030113	0.3702258	0.4422589	0.4343055	0.3783731	0.4394183	0.4899446	0.4622821	0.5697262
Subject6	0.4830201	0.6692785	0.3372974	0.5151595	0.570896	0.679136	0.6234973	0.6342146	0.5915832	0.7367966	0.7382649	0.8204807	0.704722	0.7319354
Subject7	0.5672468	0.7625005	0.2507476	0.5464565	0.650038	0.7299461	0.3938747	0.4304608	0.6280132	0.7015295	0.7143846	0.7717188	0.7019453	0.6818036
Subject8	0.704411	0.8865781	0.6356606	0.6698202	0.7152358	0.9697259	0.798193	0.7244065	0.6897086	0.7495805	0.7780223	0.8528983	0.8448703	0.9019583
Subject9	0.6202278	0.5957085	0.2330463	0.3219006	0.5495139	0.6778737	0.4146584	0.4196589	0.5532549	0.7248067	0.6107205	0.8576506	0.5372212	0.6358815
Subject10	0.6935072	0.8769212	0.36688	0.6470573	0.7305343	0.7783809	0.5787217	0.7340811	0.7308529	0.8498421	0.6298211	0.6748768	0.8126519	0.8353879
Subject11	0.8986107	1.0266233	0.7024987	0.658151	0.8075079	1.0352442	0.7817077	0.6582444	0.8662361	0.7594339	0.8997285	0.9466906	1.014702	0.9642279
Subject12	0.4781454	0.5082282	0.2570432	0.2196485	0.3824136	0.4881216	0.3819539	0.4239671	0.4821797	0.616075	0.503252	0.4672994	0.5181778	0.4985994
Subject13	0.4292502	0.694388	0.2790671	0.5549868	0.375447	0.831391	0.4231229	0.4397102	0.4901917	0.4732946	0.5317004	0.6419696	0.5477279	0.5839646
Subject14	0.3742965	0.1064527	0.2505495	0.1264261	0.533206	0.6340557	0.260735	-0.0581858	0.3239992	0.3375401	0.5808221	0.7460626	0.5499976	0.5993836
Subject15	0.2584246	0.5184941	0.0879283	0.3882419	0.3965125	0.4213553	0.1173356	0.2930538	0.2501025	0.2531056	0.4078991	0.552814	0.3185492	0.3376283
Mean value	0.549823523076923	0.677750692307692	0.355269015384615	0.469849353846154	0.588368346153846	0.7186073	0.462436846153846	0.508360307692308	0.514648407692308	0.623061076923077	0.646180507692308	0.733603746153846	0.639014269230769	0.6906108
Standard deviation (SD)	0.162910046143821	0.246748219140937	0.166917232466582	0.170887853996701	0.177562564867132	0.197661340220679	0.195407352913056	0.230010511000459	0.193328253975971	0.220480443609232	0.156300983290741	0.157918196572506	0.187784359458939	0.176898284872508
Coefficient of Variation (CV)	0.296295155274812	0.364069298551031	0.469833352300304	0.363707755683444	0.301788099288236	0.275061692555418	0.422560084773277	0.452455684521452	0.375651126256969	0.353866501656711	0.241884398291331	0.215263617996014	0.293865674838513	0.256147579609974
Session	Sedation													
Runs	Rest	Task	Rest	Task	Rest	Task	Rest	Task	Rest	Task	Rest	Task	Rest	Task
Variables and ROIs	PLE Visual		PLE SMN		PLE DAN		PLE VAN		PLE Limbic		PLE FPN		PLE DMN	
Subject1	0.7799204	1.0242744	0.5051216	0.9679534	0.6731091	1.0996398	0.527283	0.8737426	0.825299	1.1044657	0.515945	0.956232	0.6092918	0.9574044
Subject3	0.5291413	0.689177	0.1883106	0.5094777	0.6550644	0.664281	0.4637398	0.5859193	0.1970378	0.6934138	0.7636126	0.7555704	0.7473503	0.7355637
Subject5	0.7758521	0.7478022	0.4329161	0.4544319	0.6310532	0.6319764	0.4719682	0.4507639	0.4575068	0.4882077	0.5675301	0.6422665	0.6077066	0.699328
Subject6	0.8816961	0.7687125	0.7782826	0.6825979	0.7231057	0.7909505	0.7747788	0.7254253	0.5881545	0.6223918	0.7926568	0.9427253	0.8743616	0.8942593
Subject7	0.7930561	0.6287086	0.8522577	0.8186075	0.9194769	0.7421298	0.6204274	0.613636	0.1476265	0.2351175	0.8484644	0.7122606	0.8109483	0.7060119
Subject8	0.4892026	1.2018989	0.2291775	0.4458769	0.4219782	0.8838863	0.2986176	0.6248958	0.5408367	1.1149203	0.6269308	1.0112435	0.6216415	0.9355049
Subject9	0.573767	0.2023448	0.6110092	0.2343249	0.7764762	0.3006891	0.367153	0.0935302	0.3207414	0.0806994	0.6968934	0.3155087	0.7569205	0.2387691
Subject10	0.6841008	0.4058479	0.9013359	0.5443794	0.7561739	0.4913582	0.558595	0.3444568	0.4033264	0.247074	0.6260069	0.4716244	0.7067813	0.5088434
Subject11	0.6985701	0.3822638	0.2933199	0.3871831	0.6902905	0.6569199	0.2375872	0.3359071	0.0210726	0.3510305	0.6715647	0.584135	0.4578871	0.5797186
Subject12	0.5983623	0.6611942	0.7272905	0.7154212	0.7717795	0.7047827	0.7414619	0.7982797	0.4107774	0.5910795	0.7049477	0.9090195	0.750786	0.8651428
Subject13	0.0109025	0.6468837	-0.0086276	0.4456198	0.0532856	0.4868658	-0.0030097	0.4687853	0.2242593	0.8049973	0.1301074	0.5562156	0.0623726	0.5889111
Subject14	0.7064507	0.5367059	0.7180308	0.5706888	0.938545	0.7327035	0.432292	0.4254995	0.351326	0.4695188	0.5692416	0.6701664	0.5774715	0.7137904
Subject15	0.6819955	0.7011116	0.6458724	0.7026171	0.7226985	0.83102	0.472554	0.5494009	0.173767	0.2702393	0.5525609	0.6569682	0.5765081	0.7612649
Mean value	0.631001346153846	0.661301961538462	0.528792092307692	0.575321507692308	0.671772053846154	0.693631	0.458726784615385	0.530018646153846	0.358594723076923	0.544088892307692	0.6204971	0.706456623076923	0.6276944	0.706500961538462
Standard deviation (SD)	0.217426680301623	0.260756964430992	0.28299895269173	0.197034252410693	0.226024817074304	0.199472238861748	0.207738454811849	0.210431648638042	0.215339398916262	0.323772140542791	0.177763701602568	0.205748853286947	0.204366977437623	0.197380147746823
Coefficient of Variation (CV)	0.344574035581553	0.394308469650329	0.535180001381449	0.342476771294409	0.336460583288967	0.287576880015092	0.452858786055027	0.397026878516574	0.600509112539475	0.595072138248491	0.286485950703989	0.291240603550183	0.325583560149052	0.279377040502552
Session	Anesthesia													
Runs	Rest	Task	Rest	Task	Rest	Task	Rest	Task	Rest	Task	Rest	Task	Rest	Task
Variables and ROIs	PLE Visual		PLE SMN		PLE DAN		PLE VAN		PLE Limbic		PLE FPN		PLE DMN	
Subject1	-0.0230265	-0.1817359	0.0063271	-0.0913662	0.1177911	-0.0925514	0.0047453	-0.1196879	0.0060303	-0.0562481	0.0313661	-0.0747154	0.0238311	-0.0725091
Subject3	-0.3852851	-0.2701745	-0.3532975	-0.2976646	-0.5297482	-0.3968021	-0.4747472	-0.2902215	-0.2655747	-0.2215004	-0.553913	-0.4290746	-0.5472543	-0.4810505
Subject5	0.3506978	0.4863596	0.1157676	0.1464776	0.3947248	0.3956487	0.1705004	0.1923388	0.2891601	0.0382803	0.328789	0.3719488	0.3936683	0.3277592
Subject6	-0.4215499	-0.2535632	-0.1791346	-0.1746338	-0.3229597	-0.238655	-0.3978949	-0.1244045	-0.1846379	-0.0560719	-0.232875	-0.1608163	-0.2460915	-0.2095115
Subject7	-0.0175761	0.1180209	-0.2089621	-0.0585553	0.2434842	0.3581517	-0.4266571	-0.360304	-0.7774782	-0.7473796	0.1203871	0.2890181	-0.0033215	0.1137094
Subject8	-0.154926	-0.0802418	-0.1406015	-0.111272	-0.1028636	-0.0401104	-0.0748527	-0.0858547	-0.1028431	0.0085748	-0.0734006	-0.0847231	-0.0831696	-0.0888294
Subject9	-0.2515011	-0.6338028	-0.3121673	-0.613466	-0.2745857	-0.5253332	-0.226276	-0.5785332	0.2170405	-0.0225264	-0.1591476	-0.4779642	-0.133574	-0.5575456
Subject10	0.061488	0.1188542	0.0530051	0.134033	0.0275355	0.1303053	-0.093194	0.1370538	0.0925443	0.2706223	-0.0215239	0.0860663	-0.0848852	0.1165372
Subject11	0.2797599	0.2514722	0.0582222	0.0105772	0.2084702	0.1430276	0.1678192	0.0171857	-0.0114943	0.0206253	0.2365089	0.0957558	0.1953108	0.1035125
Subject12	-0.2777261	-0.0007039	-0.2493984	-0.1349629	-0.3744489	-0.0688053	-0.2714229	-0.1074981	0.1889843	0.0004403	-0.2704603	-0.0640036	-0.2640413	-0.1155018
Subject13	-0.4094977	-0.4391654	-0.4445093	-0.3652139	-0.3986619	-0.4831169	-0.3166968	-0.3168041	-0.2349192	-0.1507634	-0.3065445	-0.4709512	-0.3577949	-0.4573162
Subject14	0.2176767	0.0854766	0.2076216	0.0313837	0.1395346	0.1036407	0.15391	0.0639169	0.1456015	0.050922	0.1504279	0.0972058	0.1975285	0.0884152
Subject15	0.3196825	0.0759721	0.125628	-0.1469223	0.2959209	0.0980169	0.1821516	-0.0107625	0.1767181	-0.0098029	0.312146	0.1175415	0.2667543	0.0122493
Mean value	-0.054752584615384	-0.055633223076923	-0.101653776923077	-0.1285835	-0.044292823076923	-0.047429492307692	-0.123278084615385	-0.121813484615385	-0.035451407692307	-0.067294438461538	-0.033710761538461	-0.054208623076923	-0.049464561538461	-0.093852407692307
Standard deviation (SD)	0.285000945119535	0.298888140036971	0.20852139287104	0.207838287778439	0.306155953360394	0.296091929722939	0.245247159065767	0.217028266060752	0.286664140433236	0.233845156155908	0.265228423016899	0.273950423100799	0.267988757133092	0.267690766178061
Coefficient of Variation (CV)	-5.20525098717357	-5.37247571695969	-2.05129016533081	-1.61636825703484	-6.9120894107087	-6.24278092209133	-1.98938164744296	-1.78164401704786	-8.08611446183664	-3.47495515977238	-7.86776717323021	-5.05363183108447	-5.41779303804635	-2.85225251818448

	TW = 1 (13 self-related and 13 non-self related trials)		TW = 2 (12 self-related and 12 non-self related trials)	
**Session**	Awake			
**Variables and ROIs**	PLE Core	PLE Periphery	PLE Core	PLE Periphery
Subject1	0.8213743	0.8548659	0.8672472	0.8612869
Subject3	0.8619054	0.5832009	0.7843326	0.7527456
Subject5	0.5828322	0.5199945	0.4449643	0.3673186
Subject6	0.9099419	0.7627359	0.6823915	0.5663692
Subject7	0.6756859	0.6719368	0.80893	0.7951073
Subject8	0.8096946	0.8596826	0.9599149	0.8894593
Subject9	0.6125823	0.392586	0.7050201	0.6888512
Subject10	0.6837196	0.7385467	0.9441488	0.9811757
Subject11	0.952561	0.9353498	0.8916894	0.9348704
Subject12	0.4918526	0.4697009	0.536601	0.445048
Subject13	0.5406557	0.6387082	0.6271122	0.6705226
Subject14	0.5314293	0.1186904	0.6755685	0.2573016
Subject15	0.3617788	0.3888756	0.3295116	0.4645817
**Mean value**	0.679693353846154	0.610374938461538	0.712110161538462	0.667279853846154
**Standard deviation (SD)**	0.180099960163671	0.230708546602839	0.192397360990103	0.230935281687924
**Coefficient of Variation (CV)**	0.264972371944711	0.37797840649281	0.270179210158218	0.346084600571753

	TW = 1 (13 self-related and 13 non-self related trials)		TW = 2 (12 self-related and 12 non-self related trials)	
**Session**	Sedation			
**Variables and ROIs**	PLE Core	PLE Periphery	PLE Core	PLE Periphery
Subject1	0.9281212	0.8948391	1.0589914	1.1024138
Subject3	0.8247869	0.7371221	0.6958118	0.5911709
Subject5	0.6022391	0.5984781	0.5884739	0.5895814
Subject6	0.8814236	0.743658	0.883247	0.7781315
Subject7	0.7540226	0.7832123	0.2810008	0.3424036
Subject8	0.9528407	0.9729454	1.0353827	1.120575
Subject9	0.3355997	0.2908388	0.1163789	0.1220429
Subject10	0.3918232	0.3242174	0.5200146	0.5275125
Subject11	0.7864899	0.780376	-0.0606091	-0.2932539
Subject12	0.90645	0.7285992	0.7921092	0.656093
Subject13	0.5329199	0.3454878	0.6477604	0.5349149
Subject14	0.74289	0.6542528	0.6441215	0.4790678
Subject15	0.6338744	0.742261	0.7469386	0.7165708
**Mean value**	0.713344707692308	0.661252923076923	0.611509361538462	0.559017246153846
**Standard deviation (SD)**	0.201371361829678	0.215816379712153	0.3328196871869	0.372721117220949
**Coefficient of Variation (CV)**	0.282291800385147	0.326374934885615	0.544259349275664	0.666743503506103

	TW = 1 (13 self-related and 13 non-self related trials)		TW = 2 (12 self-related and 12 non-self related trials)	
**Session**	Anesthesia			
**Variables and ROIs**	PLE Core	PLE Periphery	PLE Core	PLE Periphery
Subject1	-0.0952366	-0.1718086	-0.0438391	-0.0861273
Subject3	-0.4637572	-0.3256538	-0.355104	-0.324862
Subject5	0.3418775	0.3559209	0.1871863	0.2395754
Subject6	-0.1266173	-0.1640679	-0.1921075	-0.2347195
Subject7	-0.0237857	0.0929533	0.0144512	0.0034496
Subject8	-0.1026622	-0.1194477	-0.0509257	-0.0432665
Subject9	-0.4904022	-0.6288342	-0.4797201	-0.5922142
Subject10	0.071648	0.0705088	0.2970683	0.2260206
Subject11	0.0939374	0.1510762	0.0873286	0.1558891
Subject12	-0.1238176	-0.0581695	-0.0366313	-0.0487846
Subject13	-0.491976	-0.5584242	-0.3581153	-0.299393
Subject14	0.072968	0.1251791	0.0459594	-0.0014468
Subject15	-0.0580818	0.0101329	0.0972463	-0.0276065
**Mean value**	-0.107377361538462	-0.093894976923076	-0.060554069230769	-0.0794989
**Standard deviation (SD)**	0.248124721319133	0.281615115327703	0.22771755317683	0.235239321363167
**Coefficient of Variation (CV)**	-2.31077312539716	-2.99925645179523	-3.76056565759449	-2.95902611687919
