# Supplementary material for: Scale-free dynamics in the core-periphery topography and task alignment decline from conscious to unconscious states
Source: Commun Biol. 2023 May 9;6:499. doi: 10.1038/s42003-023-04879-y (PMC10170069; doi:10.1038/s42003-023-04879-y)
Supplement: Supplementary file 4 — Reporting Summary [file 42003_2023_4879_MOESM4_ESM.pdf]

nature portfolio

Corresponding author(s): Philipp Klar

Last updated by author(s): Apr 21, 2023

Reporting Summary

Nature Portfolio wishes to improve the reproducibility of the work that we publish. This form provides structure for consistency and transparency in reporting. For further information on Nature Portfolio policies, see our Editorial Policies and the Editorial Policy Checklist.

Statistics

For all statistical analyses, confirm that the following items are present in the figure legend, table legend, main text, or Methods section.

n/a	Confirmed	
☐	☒	The exact sample size (*n*) for each experimental group/condition, given as a discrete number and unit of measurement
☐	☒	A statement on whether measurements were taken from distinct samples or whether the same sample was measured repeatedly
☐	☒	The statistical test(s) used AND whether they are one- or two-sided<br>*Only common tests should be described solely by name; describe more complex techniques in the Methods section.*
☒	☐	A description of all covariates tested
☐	☒	A description of any assumptions or corrections, such as tests of normality and adjustment for multiple comparisons
☐	☒	A full description of the statistical parameters including central tendency (e.g. means) or other basic estimates (e.g. regression coefficient) AND variation (e.g. standard deviation) or associated estimates of uncertainty (e.g. confidence intervals)
☐	☒	For null hypothesis testing, the test statistic (e.g. *F*, *t*, *r*) with confidence intervals, effect sizes, degrees of freedom and *P* value noted<br>*Give P values as exact values whenever suitable.*
☒	☐	For Bayesian analysis, information on the choice of priors and Markov chain Monte Carlo settings
☒	☐	For hierarchical and complex designs, identification of the appropriate level for tests and full reporting of outcomes
☒	☐	Estimates of effect sizes (e.g. Cohen's *d*, Pearson's *r*), indicating how they were calculated

Our web collection on statistics for biologists contains articles on many of the points above.

Software and code

Policy information about availability of computer code

Data collection	The assessed and analyzed functional MRI dataset was taken from a previous neuroimaging study. Please see methods section of the manuscript for details.
Data analysis	The assessed dataset was preprocessed using AFNI (ver. 22.3.06). Analyses included the softwares AFNI (ver. 22.3.06), Python (ver. 3.10.4), and Matlab (ver. 2020b).

For manuscripts utilizing custom algorithms or software that are central to the research but not yet described in published literature, software must be made available to editors and reviewers. We strongly encourage code deposition in a community repository (e.g. GitHub). See the Nature Portfolio guidelines for submitting code & software for further information.

Data

Policy information about availability of data

All manuscripts must include a data availability statement. This statement should provide the following information, where applicable:

- Accession codes, unique identifiers, or web links for publicly available datasets
- A description of any restrictions on data availability
- For clinical datasets or third party data, please ensure that the statement adheres to our policy

The functional MRI dataset assessed in this analysis is available from the corresponding author upon reasonable request.

Human research participants

Policy information about studies involving human research participants and Sex and Gender in Research.

Reporting on sex and gender	See above; we re-used a functional MRI dataset, and this dataset does not stem from a new study.
Population characteristics	See above.
Recruitment	*Describe how participants were recruited. Outline any potential self-selection bias or other biases that may be present and how these are likely to impact results.*
Ethics oversight	*Identify the organization(s) that approved the study protocol.*

Note that full information on the approval of the study protocol must also be provided in the manuscript.

Field-specific reporting

Please select the one below that is the best fit for your research. If you are not sure, read the appropriate sections before making your selection.

☒ Life sciences ☐ Behavioural & social sciences ☐ Ecological, evolutionary & environmental sciences

For a reference copy of the document with all sections, see nature.com/documents/nr-reporting-summary-flat.pdf

Life sciences study design

All studies must disclose on these points even when the disclosure is negative.

Sample size	We re-used data from 20 right-handed adults (male/female: 8/12; age 34-64 years) from a previous fMRI study.
Data exclusions	Seven out of twenty subjects had to be excluded from the fMRI analysis due to excessive head motion during scanning. We excluded subjects exhibiting more than 10% censored volumes from data analysis (motion displacement limits: translation > 0.35 mm or rotation > 3.5 °).
Replication	We controlled the empirically observed power-law exponent (PLE) results using the IRASA method and a comparison with surrogate data. Both control measurements succeeded. Additionally, we computed task-related time windows and mean frequency. Both control computations were in accordance with the PLE observations.
Randomization	*Describe how samples/organisms/participants were allocated into experimental groups. If allocation was not random, describe how covariates were controlled OR if this is not relevant to your study, explain why.*
Blinding	*Describe whether the investigators were blinded to group allocation during data collection and/or analysis. If blinding was not possible, describe why OR explain why blinding was not relevant to your study.*

Reporting for specific materials, systems and methods

We require information from authors about some types of materials, experimental systems and methods used in many studies. Here, indicate whether each material, system or method listed is relevant to your study. If you are not sure if a list item applies to your research, read the appropriate section before selecting a response.

Materials & experimental systems

n/a	Involved in the study	
☒	☐	Antibodies
☒	☐	Eukaryotic cell lines
☒	☐	Palaeontology and archaeology
☒	☐	Animals and other organisms
☒	☐	Clinical data
☒	☐	Dual use research of concern

Methods

n/a	Involved in the study	
☒	☐	ChIP-seq
☒	☐	Flow cytometry
☐	☒	MRI-based neuroimaging

Magnetic resonance imaging

Experimental design

Design type	Resting-state recordings and task recordings in three respective runs: conscious wakefulness, sedation, and anesthesia.
Design specifications	Slow event-related design: inter-trial intervals ranged from 15.5 to 25.5 seconds, jittered in 2-second steps. Each trial was presented for 0.5 seconds.
Behavioral performance measures	The conscious state was evaluated throughout the study using the Ramsay scale. Subjects were asked to strongly squeeze the investigator's hand. This procedure was applied in all three runs for awake, sedation, and under anesthesia.

Acquisition

Imaging type(s)	T1 anatomical scans and T2 functional scans.
Field strength	Siemens Magnetom 3T.
Sequence & imaging parameters	EPI, 33 slices, slice thickness = 5mm, TR = 2000ms, TE = 30ms, flip angle = 90°, field of view = 210mm^2, image matrix = 64mm^2.
Area of acquisition	Whole-brain scans.

Diffusion MRI ☐ Used ☒ Not used

Preprocessing

Preprocessing software	AFNI (ver. 22.3.06). (1) removing the first four volumes of each functional run; (2) despiking and slice timing correction; (3) co-registration with high-resolution T1-weighted anatomical images; (4) non-linear spatial normalization of the anatomical scans into MNI152 2009c space and subsequent non-linear functional to anatomical alignment (normalization); (5) functional resampling to 3x3x3mm3 voxels; (6) regression of linear and nonlinear drift (equivalent to high-pass filtering of 0.0067 Hz) plus averaging of eroded white matter (WM) and cerebrospinal fluid (CSF) signals to reduce non-neuronal noise45; (7) spatial smoothing using an 8 mm full-width at half-maximum isotropic Gaussian kernel. Volumes with head motion displacements > 0.35 mm or rotation > 3.5° were censored in both rest and task runs. We excluded subjects exhibiting more than 10% censored volumes from further data analysis.
Normalization	We applied spatial normalization of the anatomical scans into MNI152 2009c stereotactic space and subsequent non-linear functional to anatomical alignment (normalization).
Normalization template	MNI152 2009c
Noise and artifact removal	We applied regression of linear and nonlinear drift (equivalent to high-pass filtering of 0.0067 Hz) plus averaging of eroded white matter (WM) and cerebrospinal fluid (CSF) signal to reduce non-neuronal noise
Volume censoring	Volumes with head motion displacements > 0.35 mm or rotation > 3.5° were censored in both rest and task runs. We excluded subjects exhibiting more than 10% censored volumes from further data analysis.

Statistical modeling & inference

Model type and settings	No model applied.
Effect(s) tested	No task-related BOLD changes assessed.

Specify type of analysis: ☐ Whole brain ☒ ROI-based ☐ Both

Anatomical location(s)	We used previously established regions by other researchers or ROIs for our analysis (core-periphery topography).
Statistic type for inference (See Eklund et al. 2016)	No voxel-based parametric activation maps were analyzed.
Correction	We applied the Bonferroni correction whenever multiple comparisons occurred. See methods section of our manuscript for a detailed explanation.

Models & analysis

n/a	Involved in the study
☒	☐ Functional and/or effective connectivity
☒	☐ Graph analysis
☒	☐ Multivariate modeling or predictive analysis
